# Functional role of a structural water in the elevator domain of dicarboxylate transporter VcINDY

**DOI:** 10.1093/pnasnexus/pgag242

**Published:** 2026-07-16

**Authors:** Andrew Daab, Yan Li, Jennifer J Marden, Jinmei Song, David B Sauer, Da-Neng Wang, Christopher Mulligan

**Affiliations:** School of Natural Sciences, University of Kent, Canterbury, Kent CT2 7NH, United Kingdom; Department of Cell Biology, New York University School of Medicine, New York, NY 10016, USA; Department of Cell Biology, New York University School of Medicine, New York, NY 10016, USA; Department of Cell Biology, New York University School of Medicine, New York, NY 10016, USA; Centre for Medicines Discovery, Nuffield Department of Medicine, University of Oxford, Oxford OX1 3QU, United Kingdom; Department of Cell Biology, New York University School of Medicine, New York, NY 10016, USA; School of Natural Sciences, University of Kent, Canterbury, Kent CT2 7NH, United Kingdom

## Abstract

The divalent anion sodium symporter (DASS) family mediates the uptake of Krebs cycle intermediates and sulfate and influences adiposity, insulin resistance, and metabolism in mammals. While Na^+^:substrate stoichiometry is known for several DASS transporters, the location of key Na^+^-binding sites remains elusive; important information for understanding the mechanism. In VcINDY, a bacterial DASS protein, we visualized a nonprotein cryo–electron microscopy (cryo-EM) density in the middle of the transport domain. Its size and coordination suggest that it may represent either a third Na^+^ ion or a structural water molecule. Using a combination of in vitro binding and transport assays, cryo-EM structural determination, and molecular dynamic simulations, we show that the density is not a Na^+^ ion. Instead, the data indicate that the density likely represents a structural water molecule critical for transport domain integrity. Sequence and structural similarities suggest this feature may be conserved across human DASS transporters such as NaCT and NaDC3.

Significance statement Elevator-type transporters translocate substrates across the membrane via rigid-body domain movements. Their architecture is often attributed to protein–protein interactions alone. However, the role of buried water molecules, which are difficult to experimentally detect, is overlooked, despite the presence of structurally important waters in other membrane proteins. Using cryo–electron microscopy, binding, and transport measurements, this study identifies a buried structural water molecule in the bacterial sodium-dicarboxylate transporter VcINDY. The results support a role for this water molecule in maintaining the integrity of the transporter's mobile substrate-binding domain. This work highlights water molecules as integral components of membrane protein architecture and provides a framework for investigating structural waters in other transporters.

## Introduction

SLC13 transporters catalyze the Na^+^-coupled import of physiologically important anions in humans. The protein family includes NaCT (SLC13A5), NaDC1 (SLC13A2), and NaDC3 (SLC13A3) that translocate di- and tricarboxylates, and two Na^+^-sulfate cotransporters, NaS1 (SLC13A1) and NaS2 (SLC13A4) (1). SLC13 proteins have been identified in the kidneys, liver, brain, and placenta (1). Their substrates, in addition to being tricarboxylic acid (TCA) cycle intermediates, are key to several cellular processes in human physiology. The tricarboxylate citrate is a precursor and regulator of fatty acid synthesis (2), regulates fatty acid β-oxidation and glycolysis (3–5), is involved in histone acetylation (6, 7), and is a key metabolite in hepatocarcinoma cell proliferation (8). The dicarboxylate substrates, succinate and α-ketoglutarate, are important signaling molecules, regulating the fate of naive embryonic stem cells (9), and inhibiting the proliferation of malignant p53-deficient tumors, respectively (10). Sulfate-based substrates, on the other hand, are essential for the biosynthesis of sulfur-containing amino acids and influence the metabolism of glycosaminoglycans, neurotransmitters, and hormones (11–13). Due to their key roles in homeostasis and metabolism, disruption of SLC13 function is often pathogenic. Many mutations in NaCT cause early-onset epilepsy in infants (14), and NaDC3 mutations can cause acute reversible leukoencephalopathy (15). Knockouts of SLC13 orthologs in mice further strengthen the key physiological role these transporters play. Disruption of the NaCT gene protects the mice from obesity and insulin resistance and reduces cancer cell proliferation (8, 16). Deletion of NaS1 causes hyposulfatemia, hypersulfuria, seizures, reduced fertility, and growth retardation (11, 13). Furthermore, disruption of NaCT orthologs in fruit flies and nematodes results in lifespan extension through calorie restriction (17, 18). Due to their central role in homeostatic and metabolic maintenance, SLC13 family members have become attractive targets for the treatment of age-related metabolic diseases (19–22).

The SLC13 transporters belong to the larger divalent anion sodium symporter (DASS) family of transporters, which are widespread in prokaryotes and eukaryotes (23). The DASS family can be split into two mechanistically distinct clades: the Na^+^-coupled cotransporters (DASS-C) and the exchangers (DASS-E), which are Na^+^ independent (24). However, structural characterization of bacterial, yeast, and human DASS transporters (including both DASS-E and DASS-C) has revealed a well-conserved architecture; a dimeric arrangement in which each protomer consists of a scaffold domain that forms all of the interprotomer contacts, and a mobile transport domain that contains the substrate-binding sites (Fig. S1a and b) (25–29). To facilitate substrate movement across the membrane, transporters invariably undergo conformational changes between an inward-facing state (*C*_i_) and outward-facing state (*C*_o_) that alternately exposes the substrate-binding site from one side of the membrane to the other. Structural, biochemical, and computational analyses of multiple DASS family members have revealed that this alternating access is achieved via an elevator-like mechanism (30), in which the transport domain undergoes a ∼20° in-membrane rotation, a ∼35° cross-membrane tilt, and a ∼12 Å translation to transition between *C*_i_ and *C*_o_ (23, 24, 27, 28, 31, 32). Such a large, vertical movement of the transport domain also leads to the alternative exposure of part of its surface to the extramembranous aqueous environment and the hydrophobic lipid bilayer. While the transport domain displays pseudo-symmetric sequence and structural features in the N- and C-terminal halves of the protein, it remains a mystery how these two halves of the transport domain are held together during the conformational and chemical environmental changes or whether any cofactor, such as water, is involved.

In addition to the overall structure and general mechanism, the locations of the substrate-binding sites are also conserved in the DASS family. VcINDY from *Vibrio cholerae*, which is the DASS-C prototype, couples the co-transport of three Na^+^ ions to the electrogenic transport of its anionic substrate (dicarboxylates, e.g. succinate) (33, 34). VcINDY's dicarboxylate-binding site is formed by the tips of two reentrant hairpins (HP_in_ and HP_out_) and the loops from two discontinuous transmembrane helices (TM5 and TM10; Table S1 and Fig. S1b and c) (24–26, 35, 36). Structural analysis of the Na^+^ and Na^+^/substrate bound states of VcINDY reveals that two Na^+^ ions are each enclosed in a clamshell-like structure. Whereas the Na1 clamshell is formed by the L5ab loop and the tip of HP_in_, the Na2 clamshell is formed by the L10ab loop and the tip of HP_out_ (Fig. S1b). The dicarboxylate substrate is coordinated between the tips of HP_in_ and HP_out_ (24–26, 35, 36). Na^+^:substrate coupling is achieved by there being a strict requirement for the binding of Na^+^ prior to substrate binding (31, 34, 35, 37, 38). In the absence of Na^+^, VcINDY exhibits a pronounced flexibility in the substrate-binding regions, with an ensemble of polypeptide configurations precluding substrate interactions. However, Na^+^ binding selects a particular configuration that is suitable for dicarboxylate substrate binding (35). In contrast, structural characterization of the Na^+^-independent DASS-E prototype, LaINDY, reveals the presence of positively charged residues in locations equivalent to VcINDY's Na1 and Na2 sites where they function as Na^+^ ion surrogates, precluding the need for Na^+^ binding (24). In both the cotransporters and exchangers, such charge compensation in close proximity helps to achieve their respective coupling mechanisms.

The thermodynamic coupling of substrate transport to the Na^+^ gradient in DASS-C transporters is crucial to function. Understanding how Na^+^ binding influences substrate interactions and protein conformation is essential to fully delineating the mechanism. Despite this, not all of the locations of Na^+^-binding sites in the structurally characterized DASS-C cotransporters are currently known. Whereas we have shown that succinate transport is coupled to the transport of three Na^+^ ions in VcINDY (33), only two Na^+^ ions have ever been observed in the structures (Fig. S1) (24, 26, 35, 36). Similarly, in published structures of human NaCT, NaDC1, and NaDC3 (27, 29, 32), which couple the transport of both trianionic citrate and dicarboxylate to the cotransport of four and three Na^+^ ions (39), respectively, only two are visible in the structures, and they occupy equivalent positions to VcINDY's Na1 and Na2. Recent structural analysis of human NaS1, which couples the electrogenic transport of ${\mathrm{SO}}_{4}^{2-}$ to three Na^+^ ions (40), revealed a single bound Na^+^ ion located in the equivalent of VcINDY's Na1 site (27). Finally, Pho90, from *Saccharomyces cerevisiae*, which is homologous to the sulfate-specific SLC13 members, catalyzes Na^+^-driven phosphate transport (28). While the Na^+^:substrate coupling ratio is unknown for Pho90, structural analysis suggests two Na^+^ ions are bound, one in the equivalent of VcINDY's Na2 site, and the other density assigned to Na^+^ is located at a site not previously described in any DASS transporter (28).

Identification of a sodium density in an experimental map, obtained either by X-ray crystallography or cryo–electron microscopy (cryo-EM), is challenging, even at a resolution of 1.6–2.0 Å (41–44). Due to their similar scattering power (45), it is often particularly difficult to distinguish a Na^+^ ion from a water molecule buried in a protein. The situation becomes even more ambiguous when one considers the possibility that a structural water can play an essential role in the stability or integrity of the protein and the binding site is therefore conserved in the amino acid sequence (46).

Here, we describe the identification of a mystery nonprotein density in the Na^+^-succinate DASS cotransporter VcINDY that represents either a Na^+^ or structural water. Using alanine scanning mutagenesis combined with in vitro transport assays, binding affinity measurements, cryo-EM structure determination, and molecular dynamics (MD) simulations, we conclude that the observed density is likely a structural water. Based on our data, we propose that the water molecule is acting as a linchpin in the middle of the transport domain to keep the transport domain of the DASS transporters together during conformational and environmental changes of substrate translocation. Comparison with structures from other DASS transporters reveals nonprotein density in the same place, raising the possibility that this structural water-binding site is a conserved feature of the protein family.

## Results

### Nonprotein density in VcINDY cryo-EM map suggested potential Na3 or a buried water

**Figure 1:**
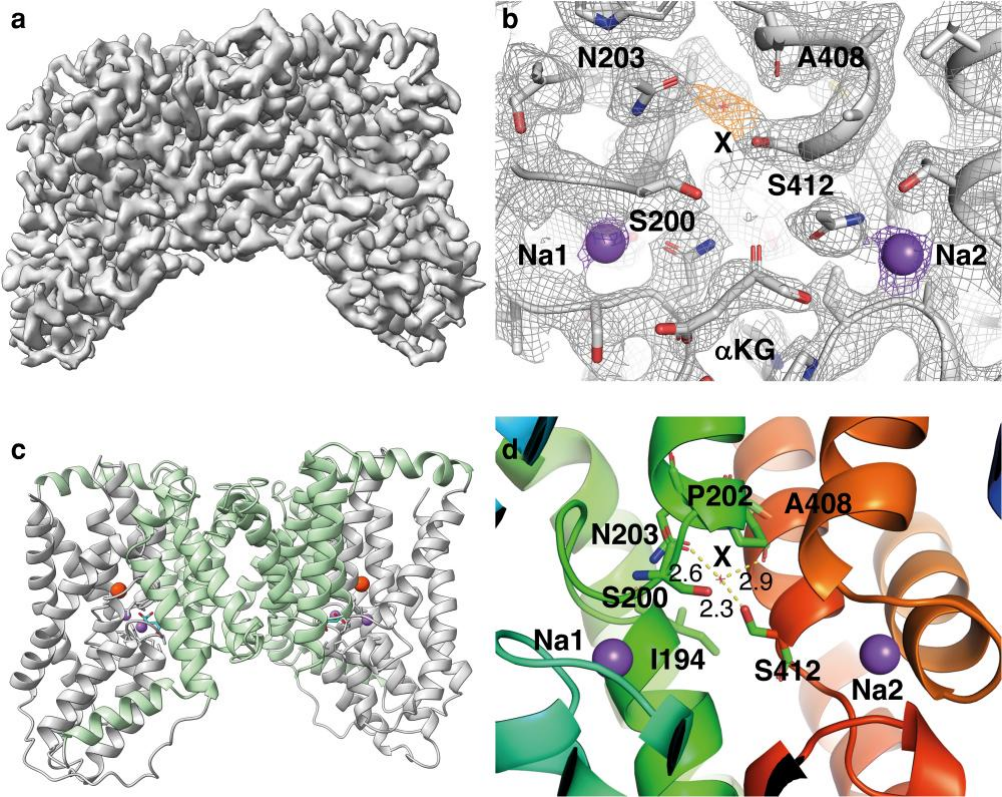
Density X in the cryo-EM map of wild-type VcINDY–Na^+^–αKG. a) A 2.35-Å 3D map of the VcINDYwt dimer in *C*_i_–*C*_i_ conformation was obtained in the presence of 100 mM NaCl and 20 mM α-ketoglutarate, in detergent LMNG. b) Local map around the Density X site in the transport domain of VcINDY. The three conserved residues among DASS-C proteins around the site, S200, N203, and S412 are labeled. The backbone oxygen of A408 also interacts with Density X. The density map is contoured at 5.5 *σ*. c) Structure model of the wild-type VcINDY *C*_i_–*C*_i_ dimer. In each protomer, the two known sodium, Na1 and Na2, are shown by purple spheres, whereas the location of Density X is indicated by an orange sphere. The scaffold and transport domains are colored light green and gray, respectively. d) Structural model around the Density X site. This density sits between the N- and C-terminal halves of the transport domain. Distances from three closest polar residues are indicated in Å. The secondary protein structures are colored by a rainbow scheme, and the αKG substrate is omitted for clarity.

After processing a new cryo-EM dataset collected from wild-type VcINDY purified in NaCl and α-ketoglutarate (αKG) to 2.35 Å resolution (Table S2 and Fig. S2), we observed that, like published VcINDYwt structures (24, 26, 35, 36), the protein dimer adopted an inward-facing *C*_i_ conformation for both protomers (Fig. 1a). We were able to assign sodium density for the two previously identified Na^+^ sites, Na1 and Na2, and the substrate density to αKG (Fig. 1b). However, unlike previous maps of 3.1–3.8 Å resolution (24, 26, 35, 36), we now observed the presence of a hitherto unseen density in the middle of the transport domain in the new 2.35 Å map that could not be assigned to a part of the protein (Figs. 1b and S3a), which we named Density X. In this map, Density X is located between the N- and C-terminal halves of the transport domain, on the periplasmic side of the dicarboxylate-binding site, forming a triangle with Na1 and Na2 (Fig. 1b–d). Density X is coordinated via polar interactions with the side chains of resides N203 (2.6 Å) and S412 (2.3 Å) and the backbone oxygen of A408 (2.9 Å, Fig. 1d). Two additional residues are at a distance of 3.9–4.0 Å, I194 and P202, and another polar residue, S200, is 4.2 Å away from Density X. Based on the spatial arrangements of the binding site, we reasoned that the side chains of N203 and S412 are essential for coordination at this site.

While the cryo-EM map at 2.35 Å resolution could not unambiguously reveal the identity of Density X, we reasoned it was likely to represent either VcINDY's missing third Na^+^ or a buried structural water molecule, which are known to stabilize transmembrane helix interactions (41, 46, 47). Multiple lines of evidence seemed to favor the former hypothesis, namely, Density X being the third Na^+^ in VcINDY. First, a multiple sequence alignment at the X site comparing Na^+^-independent DASS-E exchangers (LaINDY from *Lactobacillus acidophilus*, CitT from *Escherichia coli*, and TtdT from *E. coli*), and Na^+^-coupled DASS-C cotransporters (VcINDY, SLC13A5, and SLC13A3) provides the initial hint. While N203 at the Density X site is completely invariant in both DASS subfamilies, S412 is conserved in only the DASS-C transporters, whereas DASS-E representatives have a nonpolar residue (leucine or isoleucine) at this position (Fig. S4). Furthermore, S200 at 4.2 Å away is conservatively substituted for a threonine in NaCT and NaDC3, whereas in the exchangers, this position is an alanine or methionine (Fig. S4). Such conservation of these residues near the X site in only the Na^+^-driven DASS-C cotransporters supports a role for them in Na^+^ coupling. Second, structural comparison with other transporters with related folds is also consistent with the possibility that this is a Na^+^ ion. Previous mutagenesis studies on the non-DASS human Na^+^:phosphate transporter, NaPi-IIa, which couples the transport of ${\mathrm{HPO}}_{4}^{2-}$ to three Na^+^ ions and its transport domains share the same overall fold as VcINDY, identified equivalent residues to VcINDY's N203 and S412 as likely involved in Na^+^ coordination (48, 49). Furthermore, a single amino acid substitution (D→G) at a position equivalent to M218 in VcINDY (10.2 Å *C*_α_ to *C*_α_ from N203) was able to render NaPi-IIa electroneutral by disrupting a single Na^+^ site (50, 51), although a residue ∼10 Å away is unlikely to be directly involved in Na^+^ coordination. Finally, structural analysis of the phosphate-specific DASS cotransporter Pho90 from *S. cerevisiae* revealed a Na^+^-sized density coordinated by residues S815, N606, and S603, which are equivalent to S412, N203, and S200 in VcINDY (28), although no mutagenesis or biochemical evidence was presented to validate the sodium identity. Taken together, this evidence makes a reasonable case that Density X in our new VcINDY map belongs to a Na^+^ ion.

However, such circumstantial evidence inferred from other proteins does not directly or unambiguously resolve the identity of Density X in VcINDY as either a sodium or water within this transmembrane portion of the protein. As the scattering power of water is similar to that of Na^+^ at this resolution range (45), it is not possible to directly tell a water density from that of a sodium in a cryo-EM map at a resolution of 2.35 Å. Water molecules often play an essential role in membrane protein stability and accordingly have well-conserved binding sites. The best way to distinguish between the possibilities was to rigorously test various predictions that the two hypotheses made.

Nevertheless, we considered and dispensed with other possible identities for the atom bound at the Density X site within VcINDY. Ions with a similar size and scattering power to sodium include lithium, potassium, magnesium, calcium, and chloride. Our previous transport assays in reconstituted proteoliposomes showed that VcINDY was fully functional in the absence of lithium, potassium, magnesium, and calcium (34). In addition, when we attempted to purify VcINDY in 100 mM KCl (instead of NaCl), the protein crashed out of the solution. For chloride, the coordinating residues are unlike those one would expect for such a binding site for anions found in other chloride-binding sites (52) and the binding of a chloride is inconsistent with the stoichiometry that was determined for this transporter (34). Therefore, we consider it very unlikely that Density X represents any of these ions and instead focus on sodium and water.

### Substitution of Density X site residues did not alter Na^+^:succinate transport stoichiometry

In the absence of overwhelming evidence that Density X is a Na^+^ ion or a structural water, we proceeded to examine both hypotheses by testing their respective predictions experimentally. In the Na3 hypothesis, three residues at the binding site (N203, S412, and S200) contribute to Na^+^ coordination (S200, at 4.2 Å away in VcINDY, was included in the analysis based on the coordination of the newly proposed Na^+^ site in Pho90 (28)). This hypothesis predicts that mutating these three residues would abolish this third Na^+^ site and reduce the Na^+^:succinate stoichiometry to 2:1. As VcINDY transports succinate in its divalent form at pH 7.5 (34), such a stoichiometry change would render the transport electroneutral, as was the case for NaPi-IIa (50). The Na3 hypothesis also predicts that such mutations would reduce the Na^+^ dependence for succinate transport, which would manifest itself as an increase in the *K*_0.5_ for Na^+^. In contrast, in the buried water hypothesis, a structural water is essential for protein stability. This latter hypothesis predicts that disruption of the water site would likely (i) destabilize the protein and reduce its transport activity and (ii) leave the Na^+^ dependence of transport and VcINDY's affinity Na^+^ unchanged.

**Figure 2:**
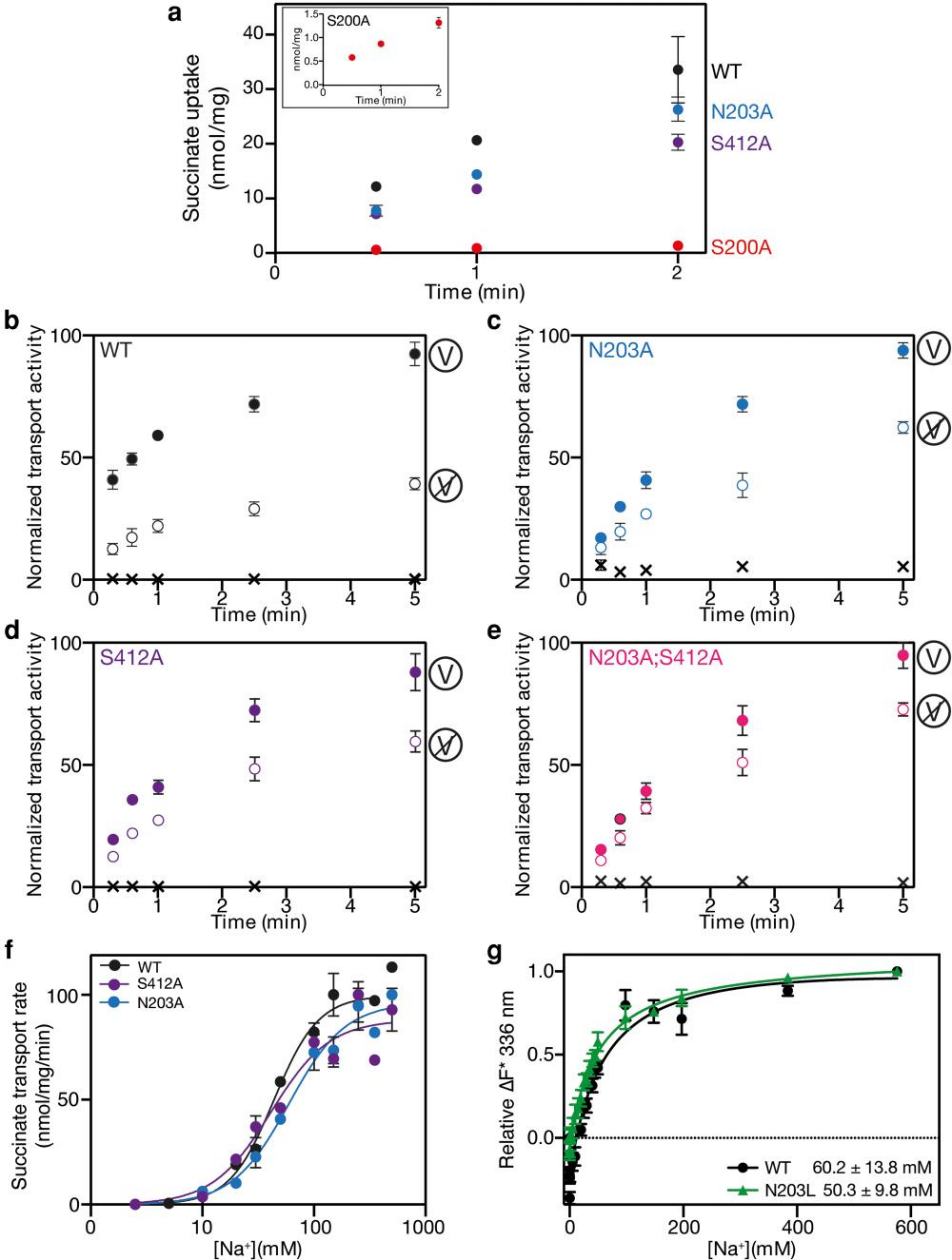
Electrogenic and kinetic properties of Density X site mutants. a) Transport of [^3^H]-succinate over time into liposomes containing wild-type (WT) and alanine mutants. The average of three datasets is shown and error bars represent SEM. Inset: Close-up of S200A data showing accumulation over time. b–e) Na^+^-driven transport of [^3^H]-succinate over time into proteoliposomes containing (b) WT, (c) N203A, (d) S412A, or (e) N203A;S412A double mutant, in the presence (filled circles) and absence (open circles) of valinomycin. Background levels of transport into protein-free liposomes (black crosses) are also shown in each graph. f) Na^+^ dependence of [^3^H]-succinate transport activity of WT, N203A, and S412A showing initial rates of succinate accumulation as a function of external Na^+^ concentration. For each panel, the average of three datasets is shown and error bars represent SEM. g) Sodium binding to WT VcINDY (black data) and N203L mutant (green data) in detergent solution as measured by intrinsic tryptophan fluorescence quenching. The average of four datasets (*n* = 4) is shown and error bars represent SEM. The *K_d_*s for Na^+^ of the WT and mutant proteins were measured and are indicated in the key.

To experimentally test predictions of the above two hypotheses, we needed to identify active VcINDY Density X–site mutants. Thus, we expressed, purified, and reconstituted the N203A, S412A and S200A mutants into liposomes and assessed the three mutants’ ability to perform Na^+^-driven succinate transport. In addition to hydrogen bonding with Density X, the first residue N203 hydrogen bonds with the backbone oxygens of I193, T196, and S200, all of which would be missing in the N203A mutant (Fig. S5a and b). S412 interacts with Density X and hydrogen bonds with the sidechain of S200 and these would not be present in the S412A mutant (Fig. S5c and d). Interestingly, mutating N203 and S412, which are closest to Density X, to an alanine resulted in robust, but reduced, transport activity compared with the wild type (Fig. 2a). This is in stark contrast to the consequences of introducing alanine mutations into VcINDY, NaCT, and NaDC3 at the Na1 or Na2 sites, which completely abolish substrate transport activity (25, 53, 54). Contrary to our observations with N203A and S412A, alanine mutation of the third residue S200, a more distant residue from the Density X site, drastically reduced VcINDY's transport activity (Fig. 2a). We speculate that this low transport activity is due to the inability of this mutant to bind substrate as analysis of VcINDY structures with various ligands reveals a key role for S200 in coordinating the ligand (Fig. S6). Due to the extremely low activity of S200A, we could not dissect this serine's contributions any further using transport assays.

Having established that both N203A and S412A near the Density X site are transport active, we next wanted to assess whether substitutions in these positions reduced the Na^+^:succinate transport ratio from 3:1 to 2:1, as would be predicted by the Na3 hypothesis. As succinate at pH 7.5 possesses a charge of −2, disruption of the Na3 site would render succinate transport electroneutral, which can be assessed by monitoring VcINDY's sensitivity to an imposed membrane potential. While transport by VcINDYwt is enhanced by the imposition of a membrane potential (negative inside compared with outside) (34), an electroneutral mutant with an abolished Na3 site and a 2:1 Na^+^:succinate stoichiometry would exhibit no change in transport activity in the presence of a membrane potential. In other words, for such electrogenic transport, the membrane potential can be viewed as a driving force in addition to the Na^+^ gradient. Therefore, when a Na^+^-site mutation abolishes sodium binding and makes the transporter electroneutral, this driving force disappears and the membrane potential no longer affects substrate uptake.

As expected, for VcINDYwt, the presence of −120 mV voltage across the proteoliposome membrane led to an increase in transport rate, compared with the absence of this membrane potential (Fig. 2b). However, we also observed transport rate enhancement for both N203A and S412A mutants (Fig. 2c and d), showing that transport remained electrogenic. These data suggest that substitution of N203 and S412 does not disrupt any Na^+^-binding sites. We reasoned that a single mutation in this area may not be sufficient to fully disrupt the binding site, so we generated the double mutant N203A:S412A. However, this double variant still remained electrogenic, suggesting that all three Na^+^ sites in the transporter remained intact (Fig. 2e). All these data are inconsistent with the Na3 hypothesis for Density X.

To rule out the possibility that merely substituting these residues for alanine was not a sufficiently disruptive change to prevent Na^+^ binding to this site, we further designed a panel of substitutions with more drastic chemical changes. To this end, we generated the following mutants: S200H, S200K, N203H, N203L, N203K, S412H, and S412R, some of which had the potential to act as surrogates for the bound Na^+^ ion similar to what is observed for the Na^+^-independent LaINDY (24). When we attempted to purify and reconstitute these mutants, we found that S200K, S412R, and S412H did not express at all. N203H expressed well and exhibited in vitro transport activity (Fig. S7a). However, this mutant retained sensitivity to the membrane potential, indicating that this substitution did not disrupt or replace Na^+^ binding (Fig. S7b). S200H also expressed well but was completely inactive in our transport assays, precluding further experiments with this variant (Fig. S7c). While inactive in our transport assays, differential scanning fluorimetry (DSF) analysis of S200H revealed a high-quality melt curve for this mutant at several NaCl concentrations, indicative of properly folded protein (Fig. S7d). However, the addition of succinate did not stabilize the protein further indicating it is binding incompetent and explaining the lack of transport activity (Fig. S7e). Both N203K and N203L exhibited substantially reduced expression, suggesting these mutations destabilized VcINDY. While these mutants were too unstable to produce interpretable DSF traces, size exclusion chromatography (SEC) analysis revealed an elution peak at the correct volume and no evidence of precipitation (Fig. S7f). Still, we obtained enough N203K and N203L for proteoliposome reconstitution, which revealed both variants to be inactive (Fig. S7g and h). Overall, most of our attempts to modify this area of VcINDY led to substantial destabilization or protein inactivation, and those substitutions that were tolerated did not render transport electroneutral.

### Substitution of Density X site residues had no effect on Na^+^ dose-dependent transport kinetics or Na^+^-binding affinity

Upon comparison of the effects of valinomycin on VcINDYwt, N203A, S412A, and the N203A:S412A double mutant, we noticed graded reductions in the magnitude of the activity enhancement in the presence of valinomycin (Fig. 2b–e). Such an effect could indicate that this site does contribute to electrogenicity by binding a charge molecule (e.g. Na^+^ ions), thus requiring further support for the lack of Na^+^ binding at this site.

As the central location of Density X is more buried than Na1 or Na2 (Fig. 1c and d), another prediction of the Na3 hypothesis was that disruption of this site would dramatically alter the Na^+^ dependence of succinate transport in VcINDY. We tested this prediction for N203A and S412A, two active alanine substitutions at the site, by monitoring their transport of [^3^H]-succinate in the presence of increasing external Na^+^ concentrations. Such measurements revealed minimal effects on the Na^+^  *K*_0.5_ for N203A and S412A compared with VcINDYwt: 61.52 ± 19.20 and 38.51 ± 11.23 mM, respectively, vs. 43.20 ± 2.22 mM for wild type (Fig. 2f). These data show that mutating N203 and S412 at the Density X site had no effect on the Na^+^ dependence of transport, consistent with our observation that mutating the Density X–binding residues did not alter the ion stoichiometry (Fig. 2b–e). Both of these observations contradict predictions made by the Na3 hypothesis. Combined, they strongly indicate that Density X is not a Na^+^ ion.

To provide further strong support that Density X is not a Na^+^ ion, we determined whether a deleterious mutation in this site (N203L) affected Na^+^-binding affinity. To compare the affinity of sodium ions to the wild-type protein and the N203L mutant, we measured Na^+^ binding to VcINDY in solution using intrinsic tryptophan fluorescence quenching (Fig. 2g). Indeed, both the wild-type VcINDY and the N203L mutant were found to bind Na^+^. Whereas the wild type binds Na^+^ with a *K*_d_ of 60.2 ± 13.8 mM, the *K*_d_ for the N203L mutant is 50.3 ± 9.8 mM. Such values of the *K*_d_ are essentially the same, again supporting the conclusion that Density X does not represent a Na^+^ ion.

### Multiple lines of evidence showed Density X is likely a structural water in VcINDY

**Figure 3:**
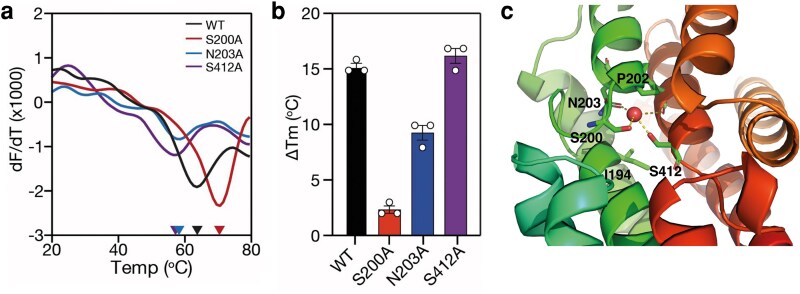
Stability of Density X–site mutants and MD simulations. a) Representative derivative (−d*F*/d*T*) plots of VcINDYwt (WT, black data) and alanine mutants S200A (red data), N203A (blue data), and S412A (purple data) in 250 mM NaCl-containing buffer. Arrows indicate the base of the troughs, which is equivalent to the melting temperature (*T*_m_). b) Succinate induced enhancement of *T*_m_ for each mutant and WT in the presence of 50 mM NaCl and 5 mM succinate (derived from derivative curves shown in Fig. S8). The average of three datasets is shown and error bars represent SEM and white circles indicates individual data points. c) Course-grained MD simulations (CGMD) of VcINDY wild type. CGMD simulation data from MemProtMD of the wild-type VcINDY embedded in a lipid bilayer, carried out in the presence of 150 mM sodium ions and flooded with water molecules. A water molecule is placed at the Density X site. The image is colored as in Fig. 1d.

Having determined that Density X is almost certainly not a Na^+^ ion, we sought to test the alternative hypothesis that Density X is a structural water molecule instead. Buried, structural water molecules are frequently observed in proteins (55–58), and in integral membrane proteins they mostly act as transmembrane helix stabilizers (46, 59, 60). Therefore, an ordered water molecule in the middle of VcINDY's transport domain would likely play a key role in the domain's structural integrity. A prediction of this buried water hypothesis was that mutations of that site would destabilize the protein. Indeed, we have already observed that S200K, S412R, and S412H mutants could not be expressed in *E. coli* cells at all, suggesting that these mutations were very destabilizing. To investigate this prediction further by a different approach, we analyzed the melting temperatures (*T*_m_) of the mutants that expressed well using DSF. Indeed, purified mutants N203A and S412A had ∼7 and ∼8 °C lower *T*_m_ than VcINDYwt, respectively (Fig. 3a, *T*_m_s of N203A, S412A, and wild type were 58.98 ± 0.33, 57.00 ± 0.88, and 65.44 ± 1.16 °C, respectively). In contrast, alanine substitution of S200, a proximal residue that does not directly interact with the presumed structural water (Fig. 1), led to a mild stabilization of ∼3.5 °C compared with wild-type VcINDY (Fig. 3a, *T*_m_ of S200A was 68.92 ± 1).

If Density X is a water molecule, then disturbance of its binding site would not affect Na^+^ binding. As predicted from the sodium-substrate coupling through sequential binding (35), the addition of succinate to VcINDY constructs with intact Na^+^ sites in NaCl solution should lead to further stabilization. Indeed, DSF analysis of N203A and S412A revealed that each mutant was stabilized by the addition of succinate in the presence of Na^+^ (Figs. 3b and S8a–d). The data reveal that while interference with this presumed water-binding site substantially destabilizes VcINDY, succinate binding still occurs, which is consistent with our previous conclusion that Na^+^ and succinate binding is unaffected. However, reflective of its inability to catalyze robust transport, the S200A mutant was only minimally stabilized upon succinate addition (Fig. 3b), suggesting this mutation compromises ligand interactions, as predicted from ligand-bound structures (Fig. S6).

These data demonstrate that modest substitutions of N203 or S412 at this tentative water site lead to substantially destabilized but active protein, but more dramatic modifications to positions N203 and S412 completely destabilize VcINDY, as shown below. However, it should be noted that even the modest substitutions of N203A, S412A, and the N203A/S412A double mutant would also disrupt multiple hydrogen bonds, which will also contribute to the relative stability of the transport domain.

To provide additional support for the presence of water in this binding site, we took advantage of preexisting atomistic MD (aMD) simulation data available in the MemProtMD database (61, 62). Conveniently, the MemProtMD pipeline provided us with aMD simulations of VcINDYwt embedded in a lipid bilayer in the presence of water and 150 mM sodium ions (62). Thus, we were able to use this existing dataset to compare binding of both of our potential ligands at physiologically relevant concentrations. Analysis of the aMD data revealed that, while water is routinely placed in the Density X position, no Na^+^ ions were found at that position (Fig. 3c), which is consistent with our functional data. This provides additional, direct support for the structural water hypothesis.

### Cryo-EM maps of the N203L mutant suggested a role for the structural water

**Figure 4:**
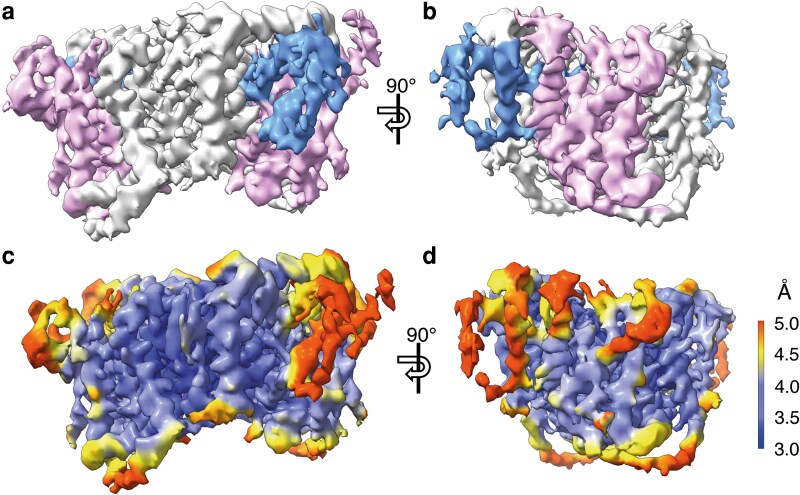
Cryo-EM maps of VcINDY–N203L–Na^+^ and sodium-binding affinity to VcINDY. a, b) Cryo-EM maps of VcINDY–N203L–Na^+^ (unsharpened), determined in 100 mM NaCl in GDN detergent. The scaffold domain is colored in gray, whereas the transport domain is colored in light pink (HP_in_, TMs 5a, 6, 10b, 11) and light blue (HP_out_, TM10a). The transport domain is broken, in the sense that the parts in pink and blue are separated. In addition, densities for parts of the transport domain (L5ab, TM5b) are missing. c, d) Local resolution maps of VcINDY–N203L–Na^+^. The maps are colored based on the local resolution, as shown in the scale bar on the left. While side chain densities are visible in the scaffold domain at the overall map resolution of 3.55 Å, the local resolution for much of the transport domain is lower, in the range of 4.5–5.0 Å, precluding model building. In particular, regions in red are highly flexible.

The central location of the water molecule between the N- and C-terminal halves of the mobile transport domain (Fig. 1) immediately suggested a possible role for the water in stabilizing the protein, perhaps by acting as a linchpin to help hold the two halves of the transport domain together during its movement in VcINDY's transport cycle. The buried water hypothesis predicts that the disruption of the water site would destabilize the protein, which we have already tested using DSF. To test this prediction further and investigate the structural consequences of disrupting this water-binding site, we obtained a nominal 3.55 Å cryo-EM map of N203L, an inactive mutant (Fig. S2b). A cryo-EM map in the presence of 100 mM NaCl in glyco-diosgenin (GDN) detergent showed a protein dimer (Fig. 4a and b), similar to the wild type (Fig. S1a), although the map quality did not allow model building. Whereas the scaffold domains remained invariant as in the wild-type protein, the transport domain is in a unique position (Fig. 4a and b), distinct from the *C*_i_ conformation of VcINDY (Fig. S9a and b) or a *C*_o_ conformation of NaDC3 (Fig. S9f and g) (32). Most notably, the transport domain appears to be distorted. While parts of the transport domain (HP_in_, TMs 5a, 6, 10b, 11) stay in a similar position to that in the *C*_o_ conformation, another part (HP_out_, TM10a) moves away by rotating ∼90° sideways (Fig. 4). Finally, densities for L5ab and TM5b, on which residue N203 is located, are missing. Such densities of a broken transport domain did not allow rigid-body model fitting. The local resolution maps also show that the transport domain of the N203L mutant is much more mobile compared with the wild-type protein (Figs. 4c, d and S9c, d). These observations fully agree with predictions made by the hypothesis that N203 coordinates a buried water molecule. Such breakage of the transport domain when the site is mutated supports the notion that the water molecule at this central location is essential for keeping the transport domain intact during the domain movement of substrate transport.

To rule out the possibility that the observed transport domain distortion was an effect of the GDN detergent on the mutant, we characterized the N203L mutant using cryo-EM in the presence of either a different detergent or a polymer. 2D classification of cryo-EM images of the N203L mutant purified in both lauryl maltose neopentyl glycol (LMNG) and amphipol yielded a similar VcINDY dimer, where the scaffold domain showed secondary structure features in 2D projections, but the density for the transport domain is weak and blurred (Figs. S9e and S10a–d). Unlike the wild-type VcINDY protein or a stable mutant, for which even a small dataset of 400–500 images from the 200 kV Arctica microscope typically yields a 3.5- to 4.0-Å 3D map with clear densities for the helices and most side chains, 3D reconstructions of the datasets from LMNG and amphipol collected on either Arctica or Krios did not yield an interpretable map in this conformation. The quality of the maps is even worse than that in GDN. As both LMNG and amphipol allow high-resolution structure determination of wild-type VcINDY (Fig. 1) (24, 35), these observations further support the notion that the transport domain of the N203L mutant is indeed less stable in various detergents than the wild-type protein.

## Discussion

In this work, we have shown that a cryo-EM density observed in the middle of the transport domain in the Na^+^-dependent dicarboxylate transporter VcINDY is likely to be a buried structural water molecule that plays a role in maintaining the integrity of the transport domain. While Density X in the cryo-EM map could represent either a sodium or a water, we tested various predictions made by either hypothesis. By combining cryo-EM, transport assays, biochemical measurements, and MD simulations, we showed that the density is unlikely to be VcINDY's missing third sodium and, on balance, the experimental evidence indicates a buried water instead.

In proteins, buried water molecules are very common. On average, one water is found every 30–50 amino acid residues, in the interior of proteins, where they often form hydrogen bonds with both main chain and side atoms (56–58, 63). The frequency of finding water in water-soluble and membrane proteins is similar (46). In membrane proteins, buried water molecules are often involved in stabilizing interactions between transmembrane α-helices, and the amino acid sequence at such a water site can be conserved.

Previously, we identified five ordered water molecules buried at the protomer–protomer interface in VcINDY dimer (35). Those water molecules form a hydrogen bond network that interacts with the transmembrane α-helices of the scaffold domains of both protomers. It was proposed that such interactions help to stabilize the VcINDY protomer–protomer interface and hence the entire dimeric architecture. Interestingly, a mutation found at the equivalent position in the water-binding pocket in the human citrate transporter NaCT, Y82C (equivalent of F92C in VcINDY), leads to an unstable dimer that has lost its transport activity (14, 29).

Here, we have identified a water molecule within the VcINDY protomer, at the interface between the two halves of the transport domain (Fig. 1), that is important to its integrity. This helps to answer a long-standing question as to how the transport domain maintains its integrity in VcINDY. This issue is particularly important for elevator-type transporters, as their large-scale vertical movements of the transport domain lead to its surface exposure to both the lipid bilayer and the extramembranous environments, requiring the domain to be especially stable. The location of the water at the interface as well as the biochemical data presented here suggest that the water molecule helps to hold the N-terminal and the C-terminal halves of the transport domain together.

In addition to Density X, there are additional unidentified densities in the cryo-EM map of wild-type VcINDY. The most prominent is a density close to Na1 (a 5.9 Å distance) and the bound αKG substrate (a 5.1 Å distance), coordinated by N151, S190, and S412 (Fig. S3b). This density is 4 Å away from the X position, and we named it Density X′. We suspect the latter density represents another water molecule. As part of the SNT signature motif in the N-terminal half of the transport domain, N151 is involved in the coordination of both Na1 and the succinate substrate. To probe the site by mutagenesis on N151 is challenging, as it will be impossible to separate the mutation's effects on X′ from those on sodium and substrate binding. If Density X′ also represents a water, more than one water molecules may help to stabilize the transporter. Mutations of S412 would affect both water molecules at X and X′. As N203L, near Density X but far away from X′, has a more deleterious effect on VcINDY, we suspect that the water at the X position is more critical for the protein's stability.

Our data reveal substantial destabilization and a lack of activity in the N203L mutant (Fig. S7), which we interpret as being a consequence of complete ablation of the water site. Interestingly, more conservative substitutions in which we replace N203 and S412 with alanine that we predict would impact the water binding have relatively mild effects on transport (Fig. 2a). We have two possible rationales for this observation; in the presence of these more conservative mutations, water-binding affinity is reduced, but as the water concentration is ∼55 M, it is still able to bind, thus permitting transport to occur. Alternatively, it is possible that multiple water molecules can bind in these mutants and compensate for the loss of side chain interactions. However, further structural work will be needed to investigate these possibilities.

In our N203L structure (Fig. 4), L5ab is missing, and as this loop forms part of the Na1 site (Fig. S1), one would expect Na^+^ binding to be affected. However, we found that this mutant was still able to bind Na^+^ with an affinity similar to wild type (Fig. 2g). We reason that this is due to these two experiments measuring VcINDY–Na^+^ interactions in different states of the transporter. Whereas binding experiments in solution measure Na^+^ binding to the inward-facing *C*_i_ state, as shown in our previous work (35), the cryo-EM maps we presented here capture the mutant structure in transition between the inward-facing *C*_i_ and outward-facing *C*_o_ state. While the ground state of VcINDY, the *C*_i_ state, can still bind water at the Density X site, it is during the conformational transition that it loses the water molecule and becomes unstable.

**Figure 5:**
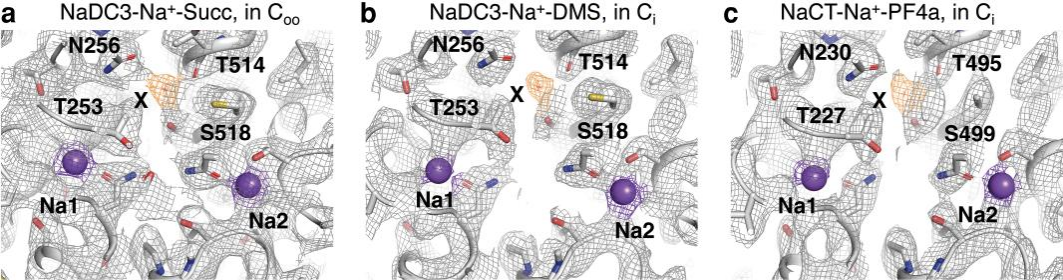
Density X observed in cryo-EM maps of the human DASS proteins NaDC3 and NaCT. a) A 2.53-Å cryo-EM map of human Na^+^-dicarboxylate cotransporter NaDC3 in sodium and succinate, in an outward-occluded *C*_oo_ conformation (EMD-42621, PDB: 8UVI). b) 2.92 Å cryo-EM map of NaDC3 obtained in sodium and 2,3-dimethylsuccinate, in an inward-open *C*_i_ conformation (EMD-42618, PDB: 8UVF). c) A 2.13-Å cryo-EM map of human Na^+^-citrate cotransporter NaCT inhibitor pF4a, in an inward open *C*_i_ conformation (EMD42614, PDB: 8UVB). The three conserved residues among DASS-C proteins around the site in each protein are labeled. Density X is colored in orange. In all three maps, the cryo-EM densities are contoured at 4.5 *σ*.

Closer reexamination of previously published cryo-EM maps of the human di-/tricarboxylate and sulfate transporters NaDC3, NaDC1, NaS1, and NaCT shows that this Density X also exists in the human SLC13 transporters (27, 29, 32, 64). The identification of the water molecule in VcINDY prompted us to ask whether the site may be conserved. To determine whether this structural water site exists in other DASS transporters, we reexamined our published cryo-EM maps of two human DASS proteins. Maps of Na^+^-dicarboxylate cotransporter NaDC3 were recently obtained in the presence of Na^+^ and succinate in several conformations (32). In maps of both outward-occluded *C*_oo_ and inward-open *C*_i_ conformations, at 2.53 and 2.92 Å resolution, respectively, Density X is observed in the same location as that in VcINDY (Fig. 5a and b). Similarly, Density X is found in the map of the Na^+^-citrate cotransporter NaCT previously reconstructed from the protein purified in Na^+^ and inhibitor PF4a (Fig. 5c). Finally, inspection of cryo-EM maps of NaDC1 (PDB8W6C/EMD37320; PDB8Y5W/EMD38954) and NaS1 (PDB8W6T/EMD37332) published by other investigators also reveals this density (27, 64). These observations, along with the amino acid sequence conservation, particularly N203 (Fig. S4), suggest a conserved structural water exists in DASS sodium cotransporters. However, further experimental work is required to confirm the identity of the density in the eukaryotic homologs.

A water molecule acting as a linchpin to hold the two halves of the transport domain together may have broad mechanistic significance for elevator-type transporters. In such proteins, the transport domain often undergoes large-scale in-membrane rotation and cross-membrane tilting and translation (23, 31, 32). As such, a transport domain typically comprises two halves found as inverted repeats in the amino acid sequence (65, 66), a water molecule acting as a linchpin is an ideal way to maintain domain integrity by helping to hold the two halves together to achieve rigid-body movement for substrate transport. This stabilization effect is particularly important during the elevator-type movement of the domain in the transport cycle, during which the transport domain changes its chemical environment. In fact, having a sodium, instead of a structural water, bound at this site would not be ideal for such a role. During the second half of the reaction cycle of DASS import, when the transporter changes from the *C*_i_-*apo* to *C*_o_-*apo* state, all the sodium sites are unoccupied. An unoccupied sodium site at the interface of the two halves of the transport domain may weaken their contact and thus the stability of the domain upon domain translocation. In contrast, a water molecule at this position does not face such a problem. The 55 M concentration of water on both sides of the membrane can ensure the site is occupied during the entire transport cycle of the transporter. To our knowledge, such a linchpin role for a water molecule has not been previously characterized in membrane transporters. Whether such a water linchpin exists in other transporter families remains to be seen.

This work still leaves the question of the location of the missing Na^+^ unanswered, despite multiple cryo-EM structures of various DASS and related proteins (24–29, 32, 35, 36). In addition to the Na^+^ ion in Pho90 at the X site in VcINDY, recent work on a TRAP transporter has suggested another possibility (67). Although not a DASS protein, the M domain of this *N*-acetylneuraminic acid transporter from *Haemophilus influenzae* shares a similar fold with the transport domain of VcINDY. In their work, the authors identified another possible metal (M) site but did not know whether the site binds a sodium. In our VcINDY maps, there is no comparable density at the equivalent position (Fig. S11). Therefore, the search for the Na3 site continues. One technical issue is that the final particle numbers used in single particle cryo-EM reconstruction are often in the range of 0.25–0.5 million. Even after considering the 10^4^ times stronger atomic scattering factors for electrons than for X-rays (45, 68), the signals for a typical cryo-EM dataset are two to three orders of magnitude lower than those of an X-ray diffraction from a small 3D crystal. Perhaps, in addition to experimental validation, we should return to the protein crystallization business and employ techniques such as difference maps in order to chase this loosely bound sodium ion.

## Methods

### Bioinformatics

Sequences for alignment were obtained from UniProt using the cart tool. The sequences chosen were functionally characterized as DASS-E or DASS-C transporters. The first three in the alignment are DASS-E transporters (named LaINDY, CitT, and TtdT) and the second set is DASS-C transporters (VcINDY, NaDC3, and NaCT). The sequences were then aligned using the Clustal Omega server (69). The alignment was imported into SnapGene Viewer for better visualization. The sequence alignment was then exported using the print function in SnapGene Viewer.

### Protein expression and purification

Wild-type VcINDY (VcINDYwt) and mutants were expressed, as previously described (38). Briefly, Lemo21 (DE3) *E. coli* are transformed with the expression plasmid pEThisINDYwt, which contains the gene encoding VcINDY in-frame with an N-terminal deca-His tag and FLAG tag (70). The expression strain is grown in PASM-5052 media in the presence of 100 µg/mL kanamycin, 25 µg/mL chloramphenicol and 250 µM L-rhamnose. Cells were grown at 37 °C until an optical density (OD) of 0.5 was reached, at which point expression was induced by the addition of 400 µM isopropyl-β-D-1-thiogalactopyranoside. Cultures were incubated overnight at 25 °C before being harvested and lysed via three passes through a cell disruptor (Avestin C3). Protein was purified as done previously (38). In summary, the membranes were resuspended in a buffer of 50 mM Tris pH 8.0, 5% glycerol, 100 mM NaCl (buffer A). To solubilize the VcINDY constructs, 20 mM DDM was added, and the solution was incubated for 40–120 min at 4 °C. After solubilization the solution was ultracentrifuged at 195,000 relative centrifugal force (rcf) for 30 min. Then, the supernatant was incubated with Talon metal affinity resin (Takara) for 16 h (overnight). The resin was washed with buffer A supplemented with 50 mM imidazole and 2 mM *n*-dodecyl-β-D-maltoside (DDM) and then washed with 20 column volumes (CV) of buffer A supplemented with 100 mM imidazole and 2 mM *n*-decyl-β-D-maltoside (DM). The protein was then eluted in buffer A with 4.3 nM trypsin, after an hour of incubation at 4 °C. A second elution was collected at the same volume in buffer A and 2 mM DDM. Sodium dodecyl sulfate–polyacrylamide gel electrophoresis gels for all proteins used in this study are presented in Fig. S12.

### Reconstitution and transport assays

VcINDY was reconstituted using a rapid dilution protocol, as described previously (37). Briefly, the 50–100 μg of protein was resuspended in a 2-mL aliquot of buffer consisting of 25 mM Tris, 100 mM NaCl, 5% glycerol, and 2 mM DM along with 3.2 g of *E. coli* Polar Lipids (Avanti). This mixture was incubated on ice for 10 min and then rapidly diluted into 65 mL of an internal buffer (20 mM Tris pH 7.5, 1 mM NaCl, and 199 mM KCl). The resultant proteoliposomes were harvested through ultracentrifugation at 158,000 rcf. The proteoliposomes underwent three freeze-thaw cycles and were stored at −80°C until used.

VcINDY activity was measured using transport assays, as described previously (34). The transport assays were conducted in a buffer containing 20 mM Tris (pH 7.5), 100 mM NaCl, 100 mM KCl, 0.9 μM unlabeled succinate, 0.1 μM ^3^H-succinate, and 1 µM valinomycin. Before commencing the transport assays, the proteoliposomes were extruded 11 times through a 400 nm filter. The assays were started with the addition of proteoliposomes (1:150 liposome volume: reaction volume) at 80 mg/mL (containing a buffer of 20 mM Tris [pH 7.5], 1 mM NaCl, and 199 mM KCl). To examine the initial transport rates, 200 μL aliquots were taken at 0.5, 1, and 2 min. The samples were then quenched in 2 mL of ice-cold buffer containing 20 mM Tris (pH 7.5) and 200 mM ChCl. The proteoliposomes were collected using a nitrocellulose filter (Cytiva part of Danaher Corp.) on a vacuum manifold and washed with a further 3 mL of the quenching buffer. The filter was dissolved, and the radioactivity was counted using 3 mL of Filter Count (Revvity) in a vial using a scintillation counter (Hidex 300 SL). For the analysis of electrogenic transport of VcINDYwt and mutants (Fig. 2b–e), the transport assays were performed as described above, but in the presence and absence of 1 µM valinomycin. Aliquots were then measured at 20, 40, 60, 150, and 300 s and were measured as previously stated. To measure the sodium dose response of the VcINDY variants, a series of sample buffers were prepared containing 2.5, 5, 10, 20, 50, 100, 150, 250, 350, and 500 mM NaCl. An aliquot was removed at 40 seconds and measured as previously stated for activity. To determine the *K*_0.5_ and reaction velocity, the activity was fitted to the Hill equation in GraphPad Prism (v 10):


$$Y=B_{\max}\times \frac{X^{h}}{\left(K_{d\;}+\;X^{h}\right)},$$

where *B*_max_ is the maximum velocity of the transport, *h* is the Hill coefficient, *K*_d_ is the dissociation-binding constant between VcINDY and sodium, and *X* is the sodium concentration.

### Differential scanning fluorimetry

DSF was performed essentially as previously described (38), with some small alterations. In brief, the VcINDY variant wasdiluted to 12 μM in DSF buffer [20 mM HEPES (4-(2-hydroxyethyl)-1-piperazineethanesulfonic acid), pH 7.5, 50 mM NaCl, 5 mM DDM]. About 5 mg/mL stock of *N*-[4-(7-diethylamino-4-methyl-3-coumarinyl)phenyl]maleimide dye was diluted 35-fold and 5 μL of the dye dilution was added to each reaction to a final reaction volume of 50 μL. Using a qPCR thermocycler (Thermo Fisher QuantStudio 3), the temperature was increased from 5 to 95 °C with a rate of 1.6 °C per second. The fluorescence of the sample was then read after every 1 °C using the SYBR Green fluorophore settings (excitation of 470 ± 15 nm and emission of 520 ± 15 nm). The *T*_m_ was determined by identifying the global minimum of the derivative curve of each reaction. The *T*_m_ was determined to be the global minimum of the derivative curve.

### Tryptophan fluorescence quenching assay to measure sodium binding

Tryptophan fluorescence quenching was used to measure the affinity of sodium to wild-type VcINDY and the VcINDY mutant N203L in detergent solution, using a protocol adapted from earlier work on VcINDY and other membrane transporters (35, 71–73). Following purification by SEC in a buffer of 25 mM Tris pH 7.5, 100 mM ChCl, and 0.05% DDM, protein was diluted to a final concentration of 4 μM in SEC buffer and used to measure sodium-binding affinity. Using a Horiba FluoroMax-4 fluorometer (Kyoto, Japan) at 22 °C and a 280-nm excitation wavelength, the emission spectrum was recorded between 290 and 400 nm. The emission maximum was determined to be 336 nm. Subsequently, the change in fluorescent emission at 336 nm was monitored with increasing concentrations of NaCl, from 0.1 to 600 mM. Each experimental condition was repeated four times. The binding curve was fit using the one-site total plus nonspecific model in GraphPad Prism:


$$Y=\frac{B_{\max}\times X}{\left(K_{d\;}+X\right)}+\mathrm{NS}\times X+\mathrm{Background},$$

where *B*_max_ is the maximum change in fluorescence, *K*_d_ is the dissociation-binding constant between VcINDY and sodium, *X* is the sodium concentration, NS is the linear, nonspecific change in VcINDY fluorescence in response to NaCl, and background is a constant for any background scattering in the experiment.

### Cryo-EM specimen preparation and image processing

For cryo-EM grid preparation, 4 μL of a protein solution at a concentration of 3–6 mg/mL was applied to a glow-discharged, 300-mesh UltrAuFoil R1.2/1.3 grid (Quantifoil, #N1-A1nAu30-01). The grid was blotted for 3–4 s under 100% humidity at 8 °C before being flash-frozen in liquid ethane using a Mark IV Vitrobot (FEI).

For VcINDYwt–Na^+^–αKG in LMNG detergent, cryo-EM data was acquired on a 300-kV Titan Krios microscope equipped with a K3 direct electron detector and an energy filter at a slit width of 20 eV, at a magnification of 105,000× and a super-resolution pixel size of 0.4125 Å (Table S2). Ice thickness was alternatively measured by zero loss peak and aperture limited scattering (74), and holes with estimated ice thicknesses of 18–30 nm were targeted for data collection. About 3,948 micrographs were collected, with defocus values ranging from −0.8 to −1.6 μm. Each micrograph was dose fractionated over 40 frames, with an accumulated dose of 53.11 e^−^ Å^−2^ in 1.8 s. All cryo-EM imaging was performed using Leginon v.3.6 (74, 75). Images were acquired with image shifts up to 7 μm, with hardware beam tilt correction enabled in Leginon. On-the-fly data quality control was performed by running MotionCor2 v1.5 (76), CTFFIND4 v4.1.13 (77), and Appion (78), picking particles using WARP v1.0.9 (79) and carrying out 2D classification cryoSPARC v3.3.1 (80). For data processing, patch contrast transfer function (CTF) estimation was performed using cryoSPARC v4.4.1 for the initial CTF parameters (80). Good particles selected from 2D classification of WARP real-time picking were used as input templates for repicking in Topaz (79, 81), and the resulting 2D projections were used as input for template picking in cryoSPARC. This yielded a total of 3,717,199 particles from all micrographs with a binned pixel size of 1.65 Å. After several rounds of 2D classification, good particles were subjected to multiple rounds of heterogeneous 3D refinement against three ab initio models generated from the selected WARP picking particles, resulting in a dataset of 897,300 particles. After re-extracting particles with a pixel size of 0.825 Å, additional rounds of heterogeneous 3D refinements were carried out. A final dataset of 274,511 particles yielded a 2.38 Å inward-open *C*_i_–*C*_i_ map of VcINDY dimer after nonuniform refinement with C2 symmetry. Following several rounds of higher-order CTF correction and nonuniform refinement, and local refinement with a soft, static mask of the protein density improved the resolution to 2.35 Å (Fig. S2 and Table S2).

For VcINDY–N203L mutant in NaCl and GDN detergent, a dataset of 1,659 micrographs was collected on a 200-kV Talos Arctica equipped with a K3 direct electron detector, at a magnification of 36,000×, with a pixel size of 0.548 Å in super-resolution mode. Defocus values ranged from −1.8 to −2.5 μm. Each micrograph was dose fractionated over 40 frames, with an accumulated dose of 44.59 e^−^ Å^−2^ in 2.4 s. For data processing, patch CTF estimation was performed using cryoSPARC v4.4.1 for the initial CTF parameters. Good particles selected from 2D classification of WARP real-time picking were used as input templates for Topaz repicking, and the resulting 2D projections were used as input for template picking in cryoSPARC. This yielded 1,567,141 particles in total from all micrographs with a binned pixel size of 2.192 Å. After several rounds of 2D classification, good particles were subjected to multiple rounds of heterogeneous 3D refinement against three ab initio models generated from the selected WARP picking particles, resulting in 295,436 particles. After re-extracting particles with a pixel size of 1.096 Å, additional rounds of heterogeneous 3D refinements were processed. 84,046 particles resulted in a final map by nonuniform refinement with C2 symmetry (Fig. S2). While the overall map resolution is 3.55 Å, the local resolution ranged 3.0–5.0 Å.

### Model building and refinement

For VcINDY–Na^+^–αKG model building, a published wild-type VcINDY structure in *C*_i_–*C*_i_ conformation (PDB ID: 7T9F) was used as the initial structural model (35). The VcINDY map was aligned against the scaffold domain of the initial model. Atomic positions in the VcINDY model were manually checked and adjusted using COOT before being refined against the corresponding map using real-space refinement in PHENIX (82, 83). All figures were prepared in Chimera X and PyMol (84, 85).

### Molecular dynamics simulations

Molecular dynamics simulation results were taken from MemProtMD's snapshot of an atomistic simulation for VcINDY (6WU3) (61).

## Supplementary Material

pgag242_Supplementary_Data

## Data Availability

The datasets generated during the current study for VcINDYwt–Na^+^–αKG are deposited in the Electron Microscopy Data Bank and Protein Data Bank as EMDB-48929 and PDB 9N5N. The source data for all graphical data are available at doi.org/10.5281/zenodo.21280193.

## References

[1] Bergeron MJ, Clémençon B, Hediger MA, Markovich D. 2013. SLC13 family of Na+-coupled di- and tri-carboxylate/sulfate transporters. Mol Aspects Med. 34:299–312. 23506872 10.1016/j.mam.2012.12.001

[2] Spencer AF, Lowenstein JM. 1962. The supply of precursors for the synthesis of fatty acids. J Biol Chem. 237:3640–3648. 13990010

[3] Garland PB, Randle PJ, Newsholme EA. 1963. Citrate as an intermediary in the inhibition of phosphofructokinase in rat heart muscle by fatty acids, ketone bodies, pyruvate, diabetes, and starvation. Nature. 200:169–170. 14073034 10.1038/200169a0

[4] Denton RM, Randle PJ. 1966. Citrate and the regulation of adipose-tissue phosphofructokinase. Biochem J. 100:420–423. 4226177 10.1042/bj1000420 PMC1265151

[5] Ruderman NB, Saha AK, Vavvas D, Witters LA. 1999. Malonyl-CoA, fuel sensing, and insulin resistance. Am J Physiol. 276:E1–E18. 9886945 10.1152/ajpendo.1999.276.1.E1

[6] Ryan DG, Frezza C, O’Neill LA. 2021. TCA cycle signalling and the evolution of eukaryotes. Curr Opin Biotechnol. 68:72–88. 33137653 10.1016/j.copbio.2020.09.014 PMC7116391

[7] Wellen KE, et al 2009. ATP-citrate lyase links cellular metabolism to histone acetylation. Science. 324:1076–1080. 19461003 10.1126/science.1164097 PMC2746744

[8] Li Z, et al 2017. Silencing of solute carrier family 13 member 5 disrupts energy homeostasis and inhibits proliferation of human hepatocarcinoma cells. J Biol Chem. 292:13890–13901. 28655760 10.1074/jbc.M117.783860 PMC5566540

[9] Carey BW, Finley LWS, Cross JR, Allis CD, Thompson CB. 2015. Intracellular α-ketoglutarate maintains the pluripotency of embryonic stem cells. Nature. 518:413–416. 25487152 10.1038/nature13981 PMC4336218

[10] Morris JP, et al 2019. α-Ketoglutarate links p53 to cell fate during tumour suppression. Nature. 573:595–599. 31534224 10.1038/s41586-019-1577-5 PMC6830448

[11] Dawson PA, Beck L, Markovich D. 2003. Hyposulfatemia, growth retardation, reduced fertility, and seizures in mice lacking a functional NaSi-1 gene. Proc Natl Acad Sci U S A. 100:13704–13709. 14578452 10.1073/pnas.2231298100 PMC263877

[12] Markovich D . 2001. Physiological roles and regulation of mammalian sulfate transporters. Physiol Rev. 81:1499–1533. 11581495 10.1152/physrev.2001.81.4.1499

[13] Markovich D . 2013. Na+–sulfate cotransporter SLC13A1. Pflügers Arch. 466:131–137. 24193406 10.1007/s00424-013-1388-8

[14] Klotz J, Porter BE, Colas C, Schlessinger A, Pajor AM. 2016. Mutations in the Na(+)/citrate cotransporter NaCT (SLC13A5) in pediatric patients with epilepsy and developmental delay. Mol Med. 22:310–321. 27261973 10.2119/molmed.2016.00077 PMC5023510

[15] Dewulf JP, et al 2019. SLC13A3 variants cause acute reversible leukoencephalopathy and α-ketoglutarate accumulation. Ann Neurol. 85:385–395. 30635937 10.1002/ana.25412

[16] Birkenfeld AL, et al 2011. Deletion of the mammalian INDY homolog mimics aspects of dietary restriction and protects against adiposity and insulin resistance in mice. Cell Metab. 14:184–195. 21803289 10.1016/j.cmet.2011.06.009 PMC3163140

[17] Rogina B, Reenan RA, Nilsen SP, Helfand SL. 2000. Extended life-span conferred by cotransporter gene mutations in *Drosophila*. Science. 290:2137–2140. 11118146 10.1126/science.290.5499.2137

[18] Fei Y-J, et al 2004. Relevance of NAC-2, an Na+-coupled citrate transporter, to life span, body size and fat content in *Caenorhabditis elegans*. Biochem J. 379:191–198. 14678010 10.1042/BJ20031807 PMC1224044

[19] Huard K, et al 2015. Discovery and characterization of novel inhibitors of the sodium-coupled citrate transporter (NaCT or SLC13A5). Sci Rep. 5:17391. 10.1038/srep17391 PMC4664966 26620127

[20] Pajor AM, et al 2016. Molecular basis for inhibition of the Na+/citrate transporter NaCT (SLC13A5) by dicarboxylate inhibitors. Mol Pharmacol. 90:755–765. 27683012 10.1124/mol.116.105049

[21] Willmes DM, et al 2018. The longevity gene INDY (I’m Not Dead Yet) in metabolic control: potential as pharmacological target. Pharmacol Ther. 185:1–11. 28987323 10.1016/j.pharmthera.2017.10.003

[22] Zahn G, et al 2022. A novel and cross-Species active mammalian INDY (NaCT) inhibitor ameliorates hepatic steatosis in mice with diet-induced obesity. Metabolites. 12:732. 36005604 10.3390/metabo12080732 PMC9413491

[23] Sauer DB, et al 2021. The ups and downs of elevator-type di-/tricarboxylate membrane transporters. FEBS J. 289:1515–1523. 34403567 10.1111/febs.16158 PMC9832446

[24] Sauer DB, et al 2020. Structural basis for the reaction cycle of DASS dicarboxylate transporters. Elife. 9:e61350. 10.7554/eLife.61350 PMC7553777 32869741

[25] Mancusso R, Gregorio GG, Liu Q, Wang DN. 2012. Structure and mechanism of a bacterial sodium-dependent dicarboxylate transporter. Nature. 491:622–626. 23086149 10.1038/nature11542 PMC3617922

[26] Nie R, Stark S, Symersky J, Kaplan RS, Lu M. 2017. Structure and function of the divalent anion/Na+ symporter from *Vibrio cholerae* and a humanized variant. Nat Commun. 8:15009. 10.1038/ncomms15009 PMC5413979 28436435

[27] Chi X, et al 2024. Cryo-EM structures of the human NaS1 and NaDC1 transporters revealed the elevator transport and allosteric regulation mechanism. Sci Adv. 10:eadl3685. 10.1126/sciadv.adl3685 PMC10980263 38552027

[28] Schneider S, Kühlbrandt W, Yildiz Ö. 2024. Complementary structures of the yeast phosphate transporter Pho90 provide insights into its transport mechanism. Structure. 32:979–988.e4. 38688287 10.1016/j.str.2024.04.005

[29] Sauer DB, et al 2021. Structure and inhibition mechanism of the human citrate transporter NaCT. Nature. 591:157–161. 33597751 10.1038/s41586-021-03230-x PMC7933130

[30] Drew D, Boudker O. 2016. Shared molecular mechanisms of membrane transporters. Annu Rev Biochem. 85:543–572. 27023848 10.1146/annurev-biochem-060815-014520

[31] Mulligan C, et al 2016. The bacterial dicarboxylate transporter VcINDY uses a two-domain elevator-type mechanism. Nat Struct Mol Bio. 23:256–263. 26828963 10.1038/nsmb.3166 PMC5215794

[32] Li Y, et al 2024. Substrate translocation and inhibition in human dicarboxylate transporter NaDC3. Nat Struct Mol Biol. 32:502–512. 39622972 10.1038/s41594-024-01433-0 PMC12645469

[33] Fitzgerald GA, Mulligan C, Mindell JA. 2017. A general method for determining secondary active transporter substrate stoichiometry. Elife. 6:e21016. 10.7554/eLife.21016 PMC5305207 28121290

[34] Mulligan C, Fitzgerald GA, Wang DN, Mindell JA. 2014. Functional characterization of a Na+-dependent dicarboxylate transporter from *Vibrio cholerae*. J Gen Physiol. 143:745–759. 24821967 10.1085/jgp.201311141 PMC4035743

[35] Sauer DB, et al 2022. Structural basis of ion—substrate coupling in the Na+-dependent dicarboxylate transporter VcINDY. Nat Commun. 13:2644. 35551191 10.1038/s41467-022-30406-4 PMC9098524

[36] Kinz-Thompson CD, et al 2026. Elevator mechanism dynamics in a sodium-coupled dicarboxylate transporter. Proc Natl Acad Sci U S A. 123:e2500723123. 10.1073/pnas.2500723123 PMC12799101 41490488

[37] Sampson CDD, Stewart MJ, Mindell JA, Mulligan C. 2020. Solvent accessibility changes in a Na+-dependent C4-dicarboxylate transporter suggest differential substrate effects in a multistep mechanism. J Biol Chem. 295:18524–18538. 33087444 10.1074/jbc.RA120.013894 PMC7939474

[38] Sampson CDD, Fàbregas Bellavista C, Stewart MJ, Mulligan C. 2021. Thermostability-based binding assays reveal complex interplay of cation, substrate and lipid binding in the bacterial DASS transporter, VcINDY. Biochem J. 478:3847–3867. 34643224 10.1042/BCJ20210061 PMC8652582

[39] Chen XZ, Shayakul C, Berger UV, Tian W. 1998. Characterization of a rat Na+-dicarboxylate cotransporter. J Biol Chem. 273:20972–20981. 9694847 10.1074/jbc.273.33.20972

[40] Busch AE, Waldegger S, Herzer T, Biber J. 1994. Electrogenic cotransport of Na+ and sulfate in *Xenopus* oocytes expressing the cloned Na+ SO4 (2−) transport protein NaSi-1. J Biol Chem. 11:385–396. 8175644

[41] Hussein R, et al 2024. Cryo-electron microscopy reveals hydrogen positions and water networks in photosystem II. Science. 384:1349–1355. 38900892 10.1126/science.adn6541

[42] Johnson ZL, Cheong C-G, Lee S-Y. 2012. Crystal structure of a concentrative nucleoside transporter from *Vibrio cholerae* at 2.4 Å. Nature. 483:489–493. 22407322 10.1038/nature10882 PMC3310960

[43] Yamashita A, Singh SK, Kawate T, Jin Y, Gouaux E. 2005. Crystal structure of a bacterial homologue of Na+/Cl− dependent neurotransmitter transporters. Nature. 437:215–223. 16041361 10.1038/nature03978

[44] Harding MM . 2002. Metal-ligand geometry relevant to proteins and in proteins: sodium and potassium. Acta Crystallogr D Biol Crystallogr. 58:872–874. 11976508 10.1107/s0907444902003712

[45] Hubbell J, McMaster W, Del Grande NK, Mallett J. International tables for X-ray crystallography. Vol. IV. Kynoch Press, Birmingham, UK, 1974. p. 45–70.

[46] Renthal R . 2008. Buried water molecules in helical transmembrane proteins. Protein Sci. 17:293–298. 18096637 10.1110/ps.073237508 PMC2222723

[47] Zhang X, et al 2020. Differential GLP-1R binding and activation by peptide and non-peptide agonists. Mol Cell. 80:485–500.e7. 33027691 10.1016/j.molcel.2020.09.020

[48] Patti M, Fenollar-Ferrer C, Werner A, Forrest LR, Forster IC. 2016. Cation interactions and membrane potential induce conformational changes in NaPi-IIb. Biophys J. 111:973–988. 27602725 10.1016/j.bpj.2016.07.025 PMC5018128

[49] Fenollar-Ferrer C, et al 2014. Structural fold and binding sites of the human Na+-phosphate cotransporter NaPi-II. Biophys J. 106:1268–1279. 24655502 10.1016/j.bpj.2014.01.043 PMC3984976

[50] Virkki LV, Forster IC, Bacconi A, Biber J, Murer H. 2005. Functionally important residues in the predicted 3(rd) transmembrane domain of the type IIa sodium-phosphate co-transporter (NaPi-IIa). J Membr Biol. 206:227–238. 16456717 10.1007/s00232-005-0796-x

[51] Bacconi A, Virkki LV, Biber J, Murer H, Forster IC. 2005. Renouncing electroneutrality is not free of charge: switching on electrogenicity in a Na+-coupled phosphate cotransporter. Proc Natl Acad Sci U S A. 102:12606–12611. 16113079 10.1073/pnas.0505882102 PMC1194947

[52] Accardi A, Lobet S, Williams C, Miller C, Dutzler R. 2006. Synergism between halide binding and proton transport in a CLC-type exchanger. J Mol Biol. 362:691–699. 16949616 10.1016/j.jmb.2006.07.081

[53] Colas C, Schlessinger A, Pajor AM. 2017. Mapping functionally important residues in the Na+/dicarboxylate cotransporter, NaDC1. Biochemistry. 56:4432–4441. 28731330 10.1021/acs.biochem.7b00503

[54] Schlessinger A, Sun NN, Colas C, Pajor AM. 2014. Determinants of substrate and cation transport in the human Na+/dicarboxylate cotransporter NaDC3. J Biol Chem. 289:16998–17008. 24808185 10.1074/jbc.M114.554790 PMC4059142

[55] Levy Y, Onuchic JN. 2004. Water and proteins: a love-hate relationship. Proc Natl Acad Sci U S A. 101:3325–3326. 14993602 10.1073/pnas.0400157101 PMC373459

[56] Carugo O . 2015. Structure and function of water molecules buried in the protein core. Curr Protein Pept Sci. 16:259–265. 25723549 10.2174/1389203716666150227162803

[57] Carugo O . 2016. Statistical survey of the buried waters in the Protein Data Bank. Amino Acids. 48:193–202. 26315961 10.1007/s00726-015-2064-4

[58] Takano K, Funahashi J, Yamagata Y, Fujii S, Yutani K. 1997. Contribution of water molecules in the interior of a protein to the conformational stability. J Mol Biol. 274:132–142. 9398521 10.1006/jmbi.1997.1365

[59] Luecke H, Schobert B, Richter HT, Cartailler JP, Lanyi JK. 1999. Structure of bacteriorhodopsin at 1.55 A resolution. J Mol Biol. 291:899–911. 10452895 10.1006/jmbi.1999.3027

[60] Marx DC, Fleming KG. 2020. Local bilayer hydrophobicity modulates membrane protein stability. J Am Chem Soc. 143:764–772. 10.1101/2020.09.01.277897 PMC8634737 33412852

[61] Newport TD, Sansom MSP, Stansfeld PJ. 2019. The MemProtMD database: a resource for membrane-embedded protein structures and their lipid interactions. Nucleic Acids Res. 47:D390–D397. 30418645 10.1093/nar/gky1047 PMC6324062

[62] Stansfeld PJ, et al 2015. MemProtMD: automated insertion of membrane protein structures into explicit lipid membranes. Structure. 23:1350–1361. 26073602 10.1016/j.str.2015.05.006 PMC4509712

[63] Denisov VP, Peters J, Hörlein HD, Halle B. 1996. Using buried water molecules to explore the energy landscape of proteins. Nat Struct Biol. 3:505–509. 8646535 10.1038/nsb0696-505

[64] Chen X, et al 2024. Structural basis for the reaction cycle and transport mechanism of human Na^+^-sulfate cotransporter NaS1 (SLC13A1). Sci Adv. 10:eado6778. 10.1126/sciadv.ado6778 PMC11584011 39576865

[65] Forrest LR . 2013. Structural biology. (Pseudo-)symmetrical transport. Science. 339:399–401. 23349276 10.1126/science.1228465

[66] Forrest LR . 2015. Structural symmetry in membrane proteins. Annu Rev Biophys. 44:311–337. 26098517 10.1146/annurev-biophys-051013-023008 PMC5500171

[67] Goyal P, et al 2024. Molecular determinants of Neu5Ac binding to a tripartite ATP independent periplasmic (TRAP) transporter. Elife. 13:RP98158. 10.7554/eLife.98158 PMC11801797 39912804

[68] Wang D-N, Kühlbrandt W. 1992. Three-dimensional electron diffraction of plant light-harvesting complex. Biophys J. 61:287–297. 19431817 10.1016/s0006-3495(92)81836-2 PMC1260246

[69] Madeira F, et al 2024. The EMBL-EBI job dispatcher sequence analysis tools framework in 2024. Nucleic Acids Res. 52:W521–W525. 38597606 10.1093/nar/gkae241 PMC11223882

[70] Love J, et al 2010. The New York Consortium on Membrane Protein Structure (NYCOMPS): a high-throughput platform for structural genomics of integral membrane proteins. J Struct Funct Genomics. 11:191–199. 20690043 10.1007/s10969-010-9094-7 PMC3099345

[71] Boulter JM, Wang DN. 2001. Purification and characterization of human erythrocyte glucose transporter in decylmaltoside detergent solution. Protein Expr Purif. 22:337–348. 11437611 10.1006/prep.2001.1440

[72] Auer M, et al 2001. High-yield expression and functional analysis of *Escherichia coli* glycerol-3-phosphate transporter. Biochemistry. 40:6628–6635. 11380257 10.1021/bi010138+

[73] Li XD, et al 2001. Monomeric state and ligand binding of recombinant GABA transporter from *Escherichia coli*. FEBS Lett. 494:165–169. 11311234 10.1016/s0014-5793(01)02334-1

[74] Rice WJ, et al 2018. Routine determination of ice thickness for cryo-EM grids. J Struct Biol. 204:38–44. 29981485 10.1016/j.jsb.2018.06.007 PMC6119488

[75] Cheng A, et al 2021. Leginon: new features and applications. Protein Sci. 30:136–150. 33030237 10.1002/pro.3967 PMC7737759

[76] Zheng SQ, et al 2017. MotionCor2: anisotropic correction of beam-induced motion for improved cryo-electron microscopy. Nat Methods. 14:331–332. 28250466 10.1038/nmeth.4193 PMC5494038

[77] Rohou A, Grigorieff N. 2015. CTFFIND4: fast and accurate defocus estimation from electron micrographs. J Struct Biol. 192:216–221. 26278980 10.1016/j.jsb.2015.08.008 PMC6760662

[78] Lander GC, et al 2009. Appion: an integrated, database-driven pipeline to facilitate EM image processing. J Struct Biol. 166:95–102. 19263523 10.1016/j.jsb.2009.01.002 PMC2775544

[79] Tegunov D, Cramer P. 2019. Real-time cryo-electron microscopy data preprocessing with warp. Nat Methods. 16:1146–1152. 31591575 10.1038/s41592-019-0580-y PMC6858868

[80] Punjani A, Rubinstein JL, Fleet DJ, Brubaker MA. 2017. cryoSPARC: algorithms for rapid unsupervised cryo-EM structure determination. Nat Methods. 14:290–296. 28165473 10.1038/nmeth.4169

[81] Bepler T, et al 2019. Positive-unlabeled convolutional neural networks for particle picking in cryo-electron micrographs. Nat Methods. 16:1153–1160. 31591578 10.1038/s41592-019-0575-8 PMC6858545

[82] Emsley P, Cowtan K. 2004. Coot: model-building tools for molecular graphics. Acta Crystallogr D Biol Crystallogr. 60:2126–2132. 15572765 10.1107/S0907444904019158

[83] Liebschner D, et al 2019. Macromolecular structure determination using X-rays, neutrons and electrons: recent developments in *Phenix*. Acta Crystallogr D Struct Biol. 75:861–877. 31588918 10.1107/S2059798319011471 PMC6778852

[84] Pettersen EF, et al 2021. UCSF ChimeraX: structure visualization for researchers, educators, and developers. Protein Sci. 30:70–82. 32881101 10.1002/pro.3943 PMC7737788

[85] The PyMOL Molecular Graphics System, Version 3.0 . Schrödinger, LLC.

