# Supplementary material for: Functional role of a structural water in the elevator domain of dicarboxylate transporter VcINDY
Source: PNAS Nexus. 2026 Jul 16;5(7):pgag242. doi: 10.1093/pnasnexus/pgag242 (PMC13394699; doi:10.1093/pnasnexus/pgag242)
Supplement: pgag242_Supplementary_Data [file pgag242_supplementary_data.pdf]

Supplementary information

Supplementary Table 1. Previously published VcINDY structures

**Protein / substrate**	**Method**	**PDB ID**	**Resolution**	**Reference**
VcINDY-Na^+-Citrate	X-ray	4F35	3.2 Å	Mancusso *et al*.[1]
VcINDY-Na^+-Succinate	X-ray	5UL7	2.8 Å	Nie *et al*.[2]
VcINDY-Na^+-Citrate	X-ray	5UL9	2.78 Å	Nie *et al*.[2]
VcINDY-Na^+-Citrate (Humanized mutant)	X-ray	5ULD	2.78 Å	Nie *et al*.[2]
VcINDY-Na^+-Succinate (Humanized mutant)	X-ray	5ULE	2.8 Å	Nie *et al*.[2]
VcINDY-Na^+-Fumarate	X-ray	6OKZ	3.29 Å	Kinz-Thompson *et al*.[3]
VcINDY-Na^+-Malate	X-ray	6OL0	3.5 Å	Kinz-Thompson *et al*.[3]
VcINDY-Na^+-Succinate	X-ray	6OL1	3.09 Å	Kinz-Thompson *et al*.[3]
VcINDY-Na^+-Terephthalate	X-ray	6WXT	3.92 Å	Sauer *et al*.[4]
VcINDY-100 mM Na^+ (Amphipol)	Cryo-EM	6WU3	3.16 Å	Sauer *et al*.[4]
VcINDY-100 mM Na^+-Fab84 (Nanodisc)	Cryo-EM	6WW5	3.15 Å	Sauer *et al*.[4]
VcINDY-*apo* (Amphipol)	Cryo-EM	7T9F	3.23 Å	Sauer *et al*.[5]
VcINDY-300 mM Na^+ (Amphipol)	Cryo-EM	7T9G	2.83 Å	Sauer *et al*.[5]

Supplementary Table 2. Cryo-EM data collection, model refinement and validation statistics of VcINDYwt

	**VcINDYwt-Na$^+$-αKG** (EMDB-48929) (PDB 9N5N)
**Data collection**	
Magnification (x)	105,000
Voltage (kV)	300
Electron exposure (e$^-$/Å^2)	53.11
Defocus range (μm)	-0.7 to -1.9
Pixel size (Å)	0.4125
Symmetry imposed	C2
Initial particle number	3,717,199
Final particle number	274,511
Map resolution (Å)	2.35
FSC threshold	0.143
Map resolution range (Å)	2.1-2.7
**Refinement**	
Initial model used (PDB code)	7T9F
Model resolution (Å)	2.6
FSC threshold	0.5
Model resolution range (Å)	n/a
Map sharpening *B* factor (Å^2)	-86
Model composition	
Non-hydrogen atoms	7,004
Protein residues	924
Ligands	H_2O:2/αKG:2
Ions	Na^+:4
B factors (Å^2)	
Protein	52.28
Ligand	55.28
R.m.s. deviations	
Bond lengths (Å)	0.002
Bond angles (°)	0.519
Validation	
MolProbity score	1.3
Clashscore	5.54
Poor rotamers (%)	0.00
Ramachandran plot	
Favored (%)	99.13
Allowed (%)	0.87
Disallowed (%)	0.00

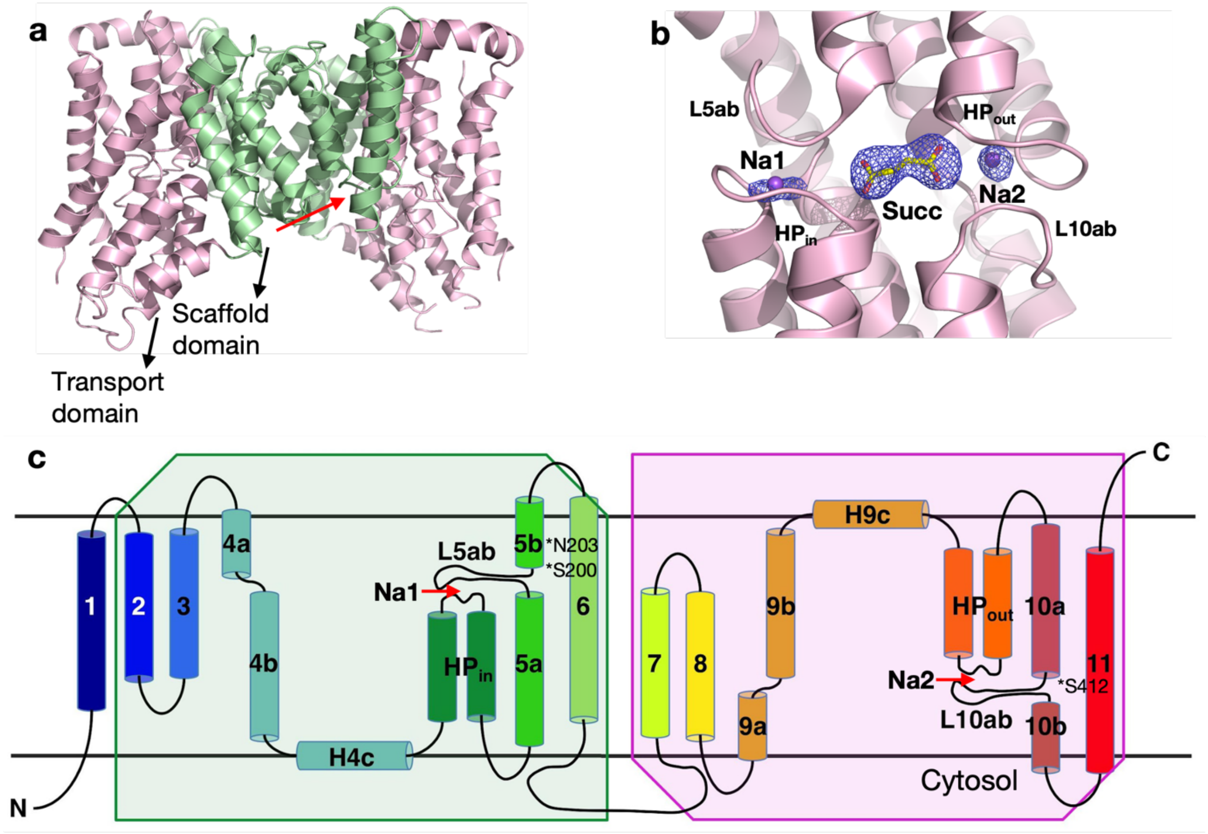

Supplementary Fig. 1. Structure of wildtype VcINDY in complex with sodium and succinate (Succ). **a** Domain structure of the transporter dimer. In each protomer, the scaffold domain is colored light green, whereas the transport domain is colored light pink. The red arrow indicates the direction of the view in **b**. **b** Substrate binding site in VcINDY. The electron density map for the succinate (Succ) and Na^+s (Na1 and Na2) are shown in mesh. The figures are generated from previously deposited VcINDY-Na^+-Succ structure determined by X-ray crystallography (PDB ID: 6OL1)[3]. **c** Transmembrane topology of VcINDY. The locations of Na1 and Na2 are indicated by red arrows, and the positions of S200, N203 and S412 are labeled.

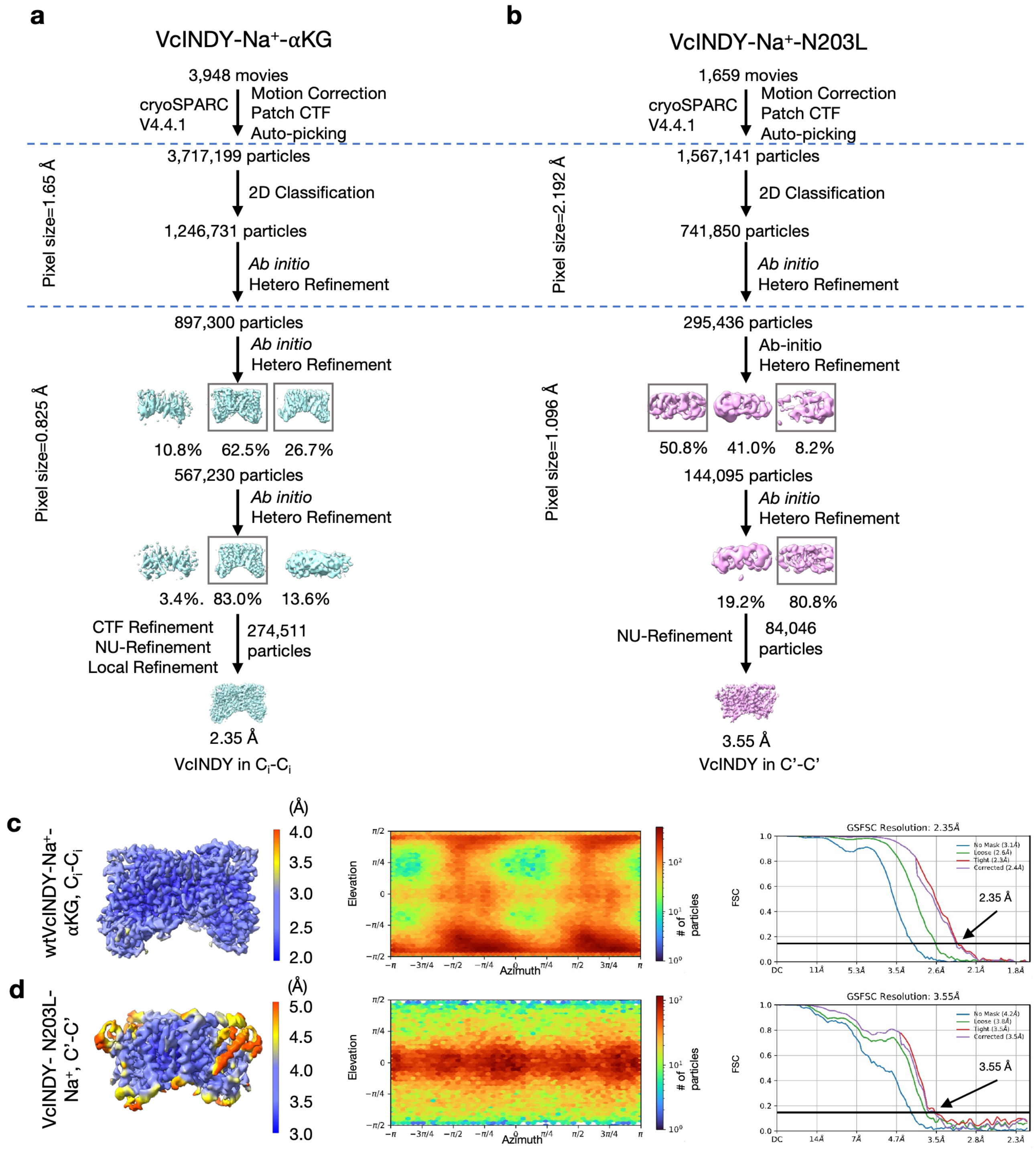

Supplementary Fig. 2. Flow chart for cryo-EM structure determination of VcINDY. **a** Image process procedure for the VcINDYwt-Na^+-αKG dataset. **b** Image process procedure for the VcINDY-N203L-Na^+ dataset. The map obtained yielded a conformation C' that is neither an inward- (C_i) nor outward (C_o) conformation. We interpret the map represents a broken transporter protein. **c** Local resolution map, particle angular distribution and FSC curves of the VcINDYwt-Na^+-αKG dataset. **d** Local resolution map, particle angular distribution and FSC curves of the VcINDY-N203L-Na^+ dataset.

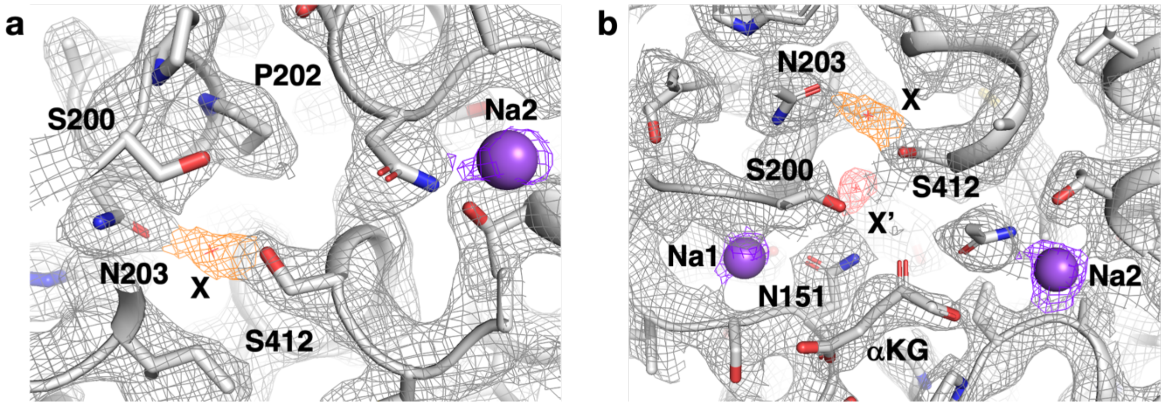

Supplementary Fig 3. Cryo-EM maps of VcINDYwt. a Cryo-EM map of VcINDYwt around Density X, viewed at a 70° angle from that in Fig. 1b. **b** Cryo-EM map of VcINDYwt showing an additional unassigned density, Density X'. The two densities are 4.0 Å from each other, and they share a coordination residue S412.

```
                                                             S200 N203
LaINDY          -----------YDSKPNDPSRKKIGAYLDFMAFHANILSTALFITGAAPNLVAQQ-MAA-     221
EcCitT          -----------FKSFPNDPSARRIGGYLMWMMVISTSLSSSMFVTGAAPNVLGLEFVSK-     225
EcTtdT          -----------YQSQPNDSSSRSIGSYIMWMGIVADCVTSAIFLTAMAPNLLLIGLMKS-     224
VcINDY          ---------------------QRSTYVFVLLGVAYSASIGGIATLVGSPPNAIAAAEV---     210
hNaCT           PGS-QVIFEGPTLGQQEDQERKRLCKAMTLCICYAASIGGTATLTGTGPNVVLLGQMNEL     240
hNaDC3          PGETEVPLDLPADSRKEDEYRRNIWKGFLISIPYSASIGGTATLTGTAPNLILLGQLKSF     266
                                     :     .      :  :     :..  ** :    :

                                          S412
LaINDY          PFLTVATAM-GAPLGLSAMLLAFTGVINASTTHYANGPASILATTGYVKQSEWWKMNFIL     466
EcCitT          VILAVGKGIPGVPMEQLCILLVLSIGIMGCLTPYATGPGVIIYGCGYVKSKDYWRLGAIF     467
EcTtdT          MMIAAALAMPEIPLPVFCLMVGAAIGLGSILTPYATGPSPIYYGSGYLPTADYWRLGAIF     466
VcINDY          VFATVAEAFGMSPVL--LSVLIAVAASCAFMLPVATPPNAIVFASGHIKQSEMMRVGLYL     445
hNaCT           IFASMSRSIGLNPLY--IMLPCTLSASFAFMLPVATPPNAIVFTYGHLKVADMVKTGVIM     532
hNaDC3          VLAELAIRLRVHPLY--LMIPGTVGCSFAFMLPVSTPPNSIAFASGHLLVKDMVRTGLLM     551
                 :   .  :   *:     :        .     :. *  *    *::   :  : .  :
```

Supplementary Fig. 4. Sequence alignment of DASS proteins. Amino acid sequences were aligned using a Clustal Omega server[6]. Three representative sequences were chosen from known DASS-E and DASS-C transporters (DASS-C [EcCitT, EcTtdT, LaINDY] and DASS-E [VcINDY, NaDC3, NaCT]). S200, N203 and S412 (VcINDY numbering) are highlighted and labeled.

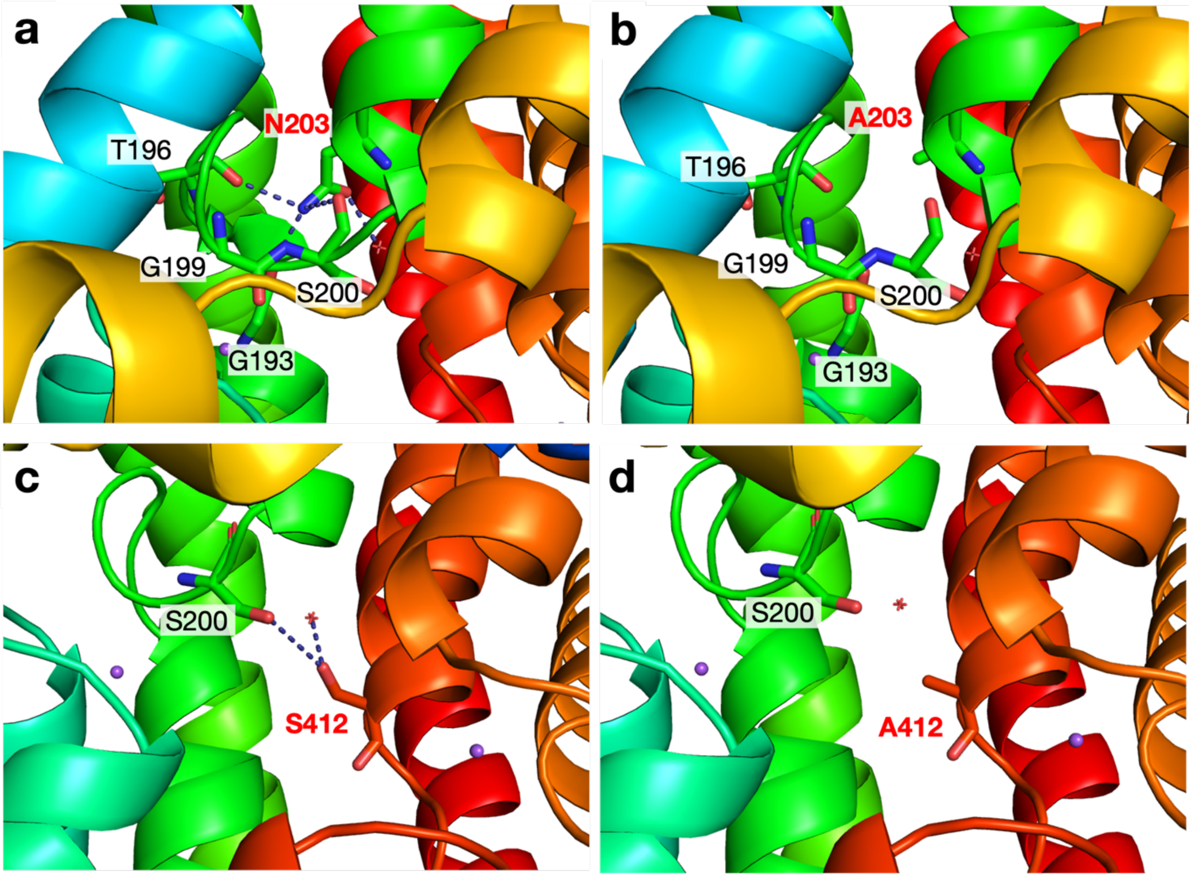

Supplementary Fig 5. Structural consequences of mutating N203 and S412. Cartoon representation of VcINDYwt-Na^+-αKG centered on the interactions of **a** N203, **b** A203 (in the N203A mutant), **c** S412, and **d** A412 (in the S412A mutant). Hydrogen bonds are indicated by dashed black lines. N203 sidechain interacts with Density X and the backbone oxygens of G193, T196 and S200 **(a)**, all of which are missing when N203 is mutated to alanine **(b)**. S412 hydrogen bonds with Density X and with the sidechain of S200 **(c)**, both of which are missing when mutated to alanine **(d)**.

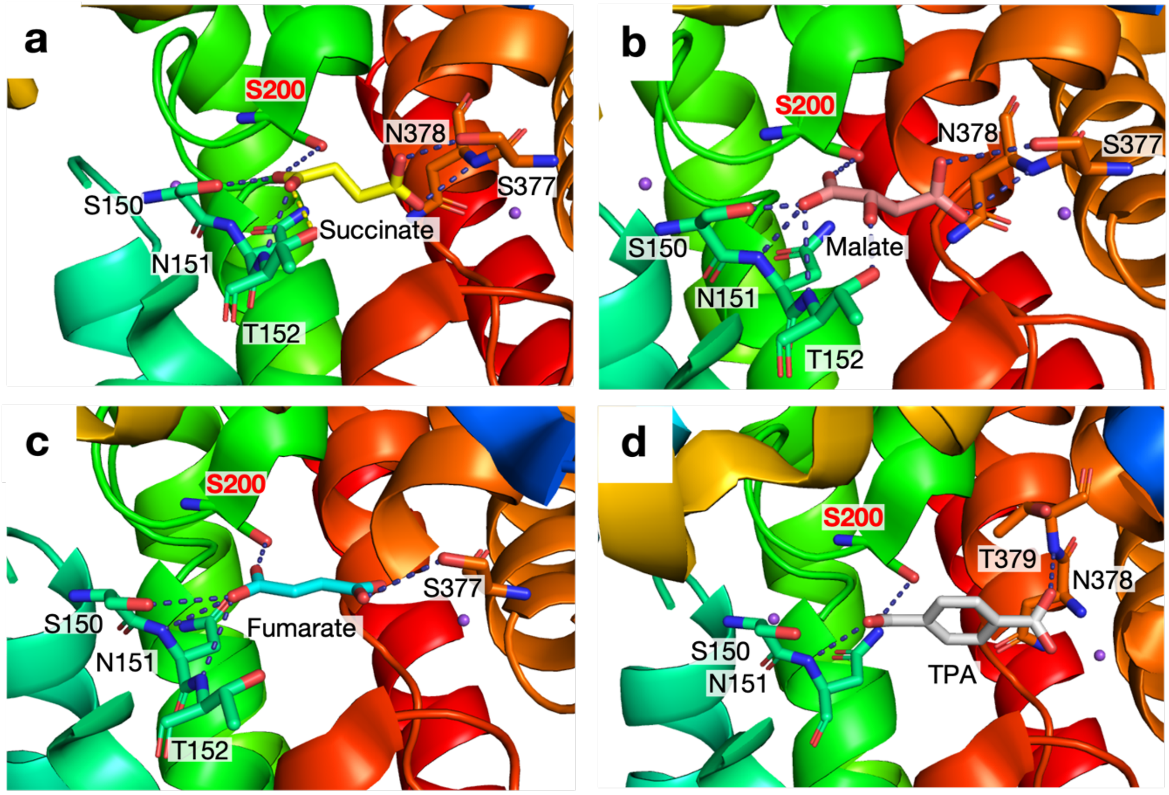

Supplementary Fig 6. Interaction between S200 and different VcINDY ligands. Cartoon representation of VcINDY structures in the presence of **a** Succinate (PDB: 6OL1), **b** Malate (PDB: 6OL0), **c** Fumarate (PDB: 6OKZ), **d** Terephthalate (TPA, PDB: 6WTZ). Hydrogen bonds are indicated by dashed black lines. Residue numbers are indicated and S200 is highlighted in red.

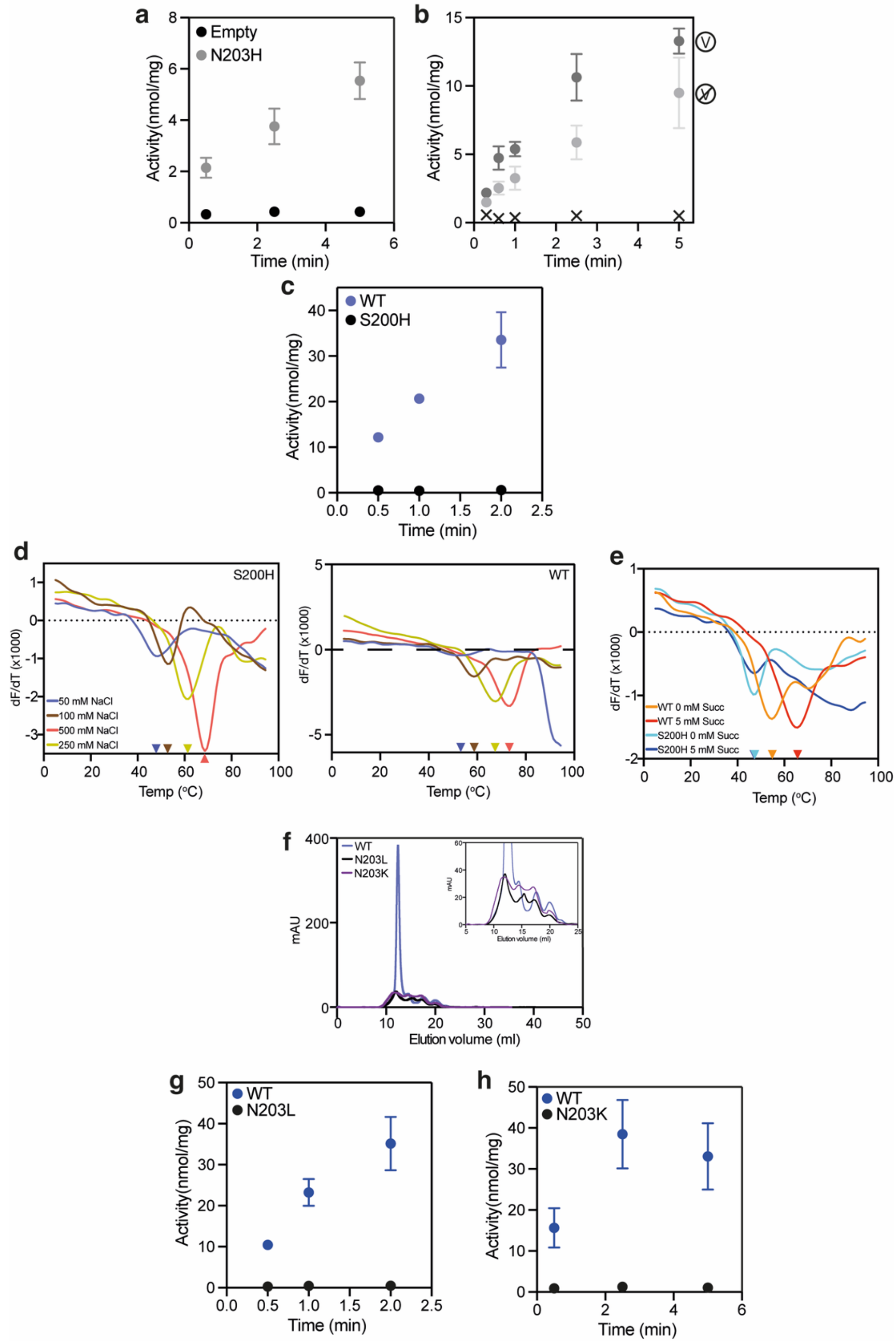

Supplementary Fig. 7. Transport activity of Density X site mutants. a Transport of [^{3}H]-succinate over time into liposomes containing WT and N203H mutant. **b** Na^+-driven transport of [^{3}H]-succinate over time into proteoliposomes containing N203H in the presence (dark gray) and absence (light gray) of valinomycin. Background levels of transport into protein-free liposomes (black crosses) are also shown. **c** Transport of [^{3}H]-succinate over time into liposomes containing WT and S200H. **d** DSF analysis of S200H and WT with increasing concentrations of NaCl. **e,** DSF analysis of S200H and WT in the presence of 50 mM NaCl with and without addition of 5 mM succinate. **f** SEC traces of WT, N203L and N203K, *inset,* magnified view of N203L and N203K traces. **g, h** Transport of [^{3}H]-succinate over time into liposomes containing WT and N203L (**g**), or N203K (**h**). Average of 3 data sets are shown and error bars represent SEM.

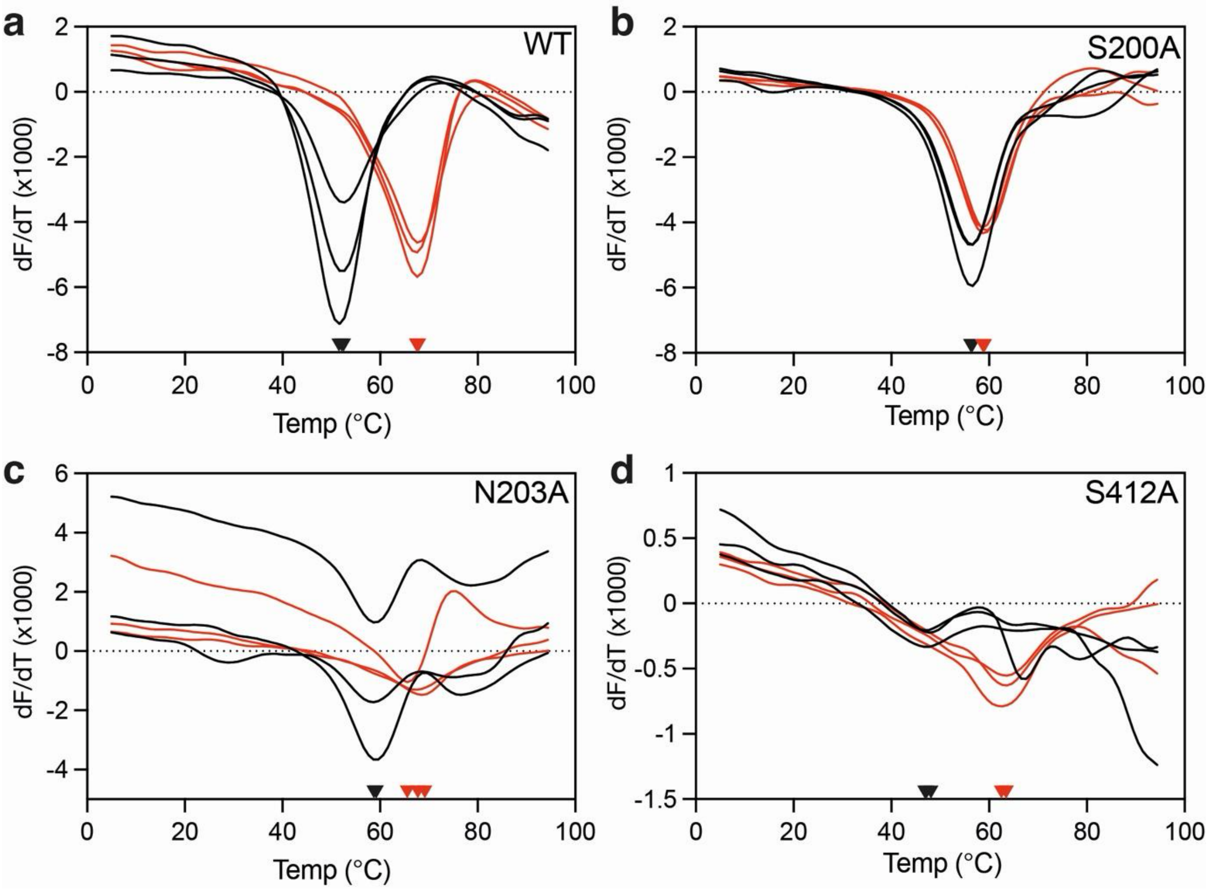

Supplementary Fig. 8. Succinate-induced stabilization of VcINDYwt and mutants. Derivative (-dF/dT) plots of **a** VcINDYwt, **b** S200A, **c** N203A and **d** S412A in the presence (red data) and absence (black data) of 5 mM succinate. All melt curves were collected in the presence of 50 mM NaCl, except for N203A which was collected in 250 mM NaCl to improve data quality to determine the succinate induced ΔTm. Arrows indicate the base of the troughs, which is equivalent to the melting temperature (Tm).

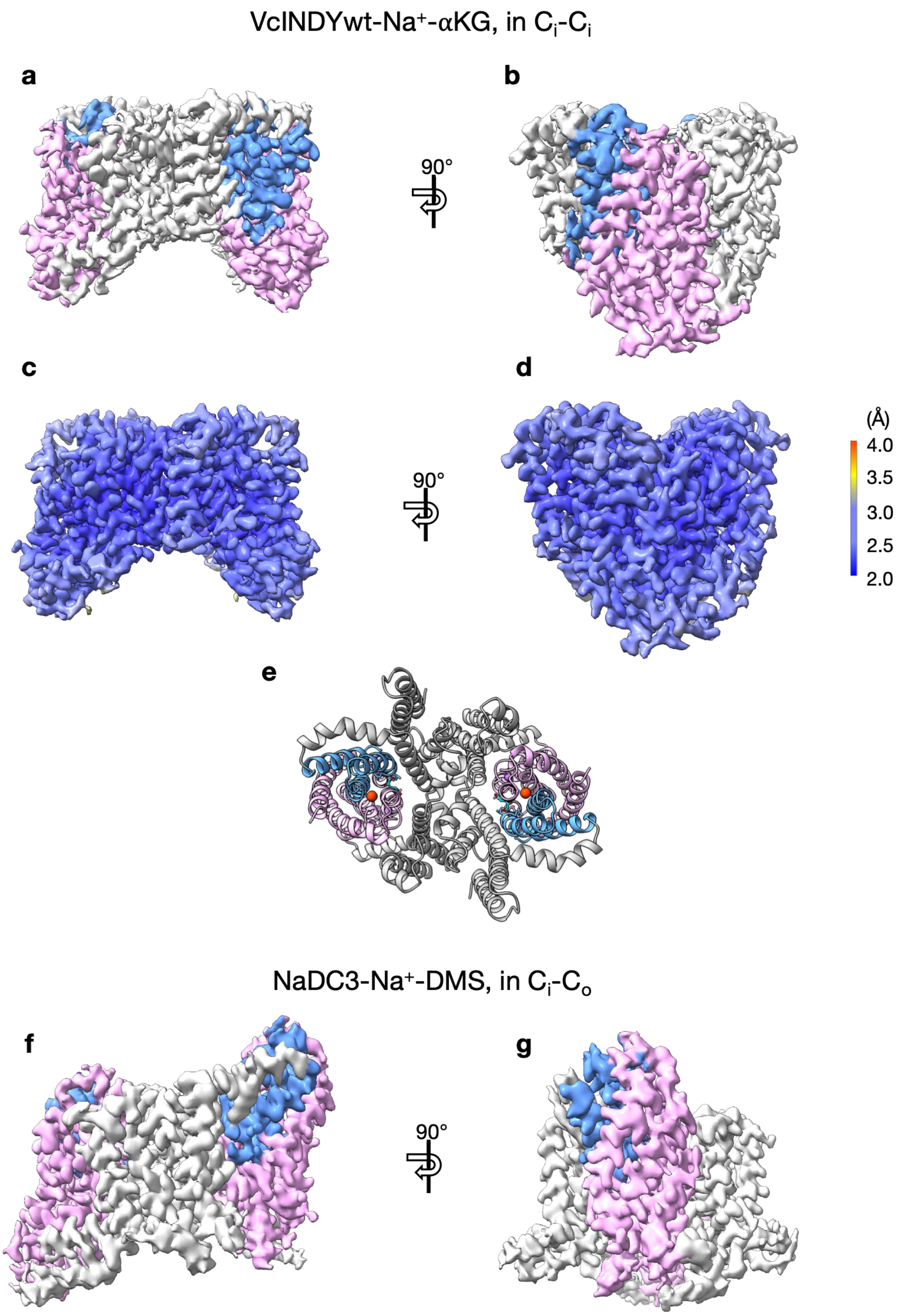

Supplementary Fig. 9 Cryo-EM of VcINDYwt and NaDC3. a,**b** Cryo-EM maps of VcINDYwt-Na$^+$-αKG in C_i-C_i conformation, colored in the same way as in Fig. 4a,b. The scaffold domain is colored in grey, whereas the transport domain is colored in light pink (HP_{in}, TMs 5a, 6, 10b, 11) and light blue (HP_{out},TM10a). **c**,**d** Local resolution maps of VcINDYwt-Na$^+$-αKG determined in the current work. While the overall resolution of the map is at 2.35 Å, the local resolution for most of the regions are in the range of 2.0 - 2.5 Å. **e** Structural model of VcINDYwt, viewed from the extracellular space. Colored the same as (a). The position of Density X is indicated with an orange sphere. **f**,**g** Cryo-EM maps of human NaDC3-Na$^+$-DMS (DMS: dimethyl-succinate) in C_i-C_o conformation (EMDB: 8UVG), colored in the same way as in **a**,**b**.

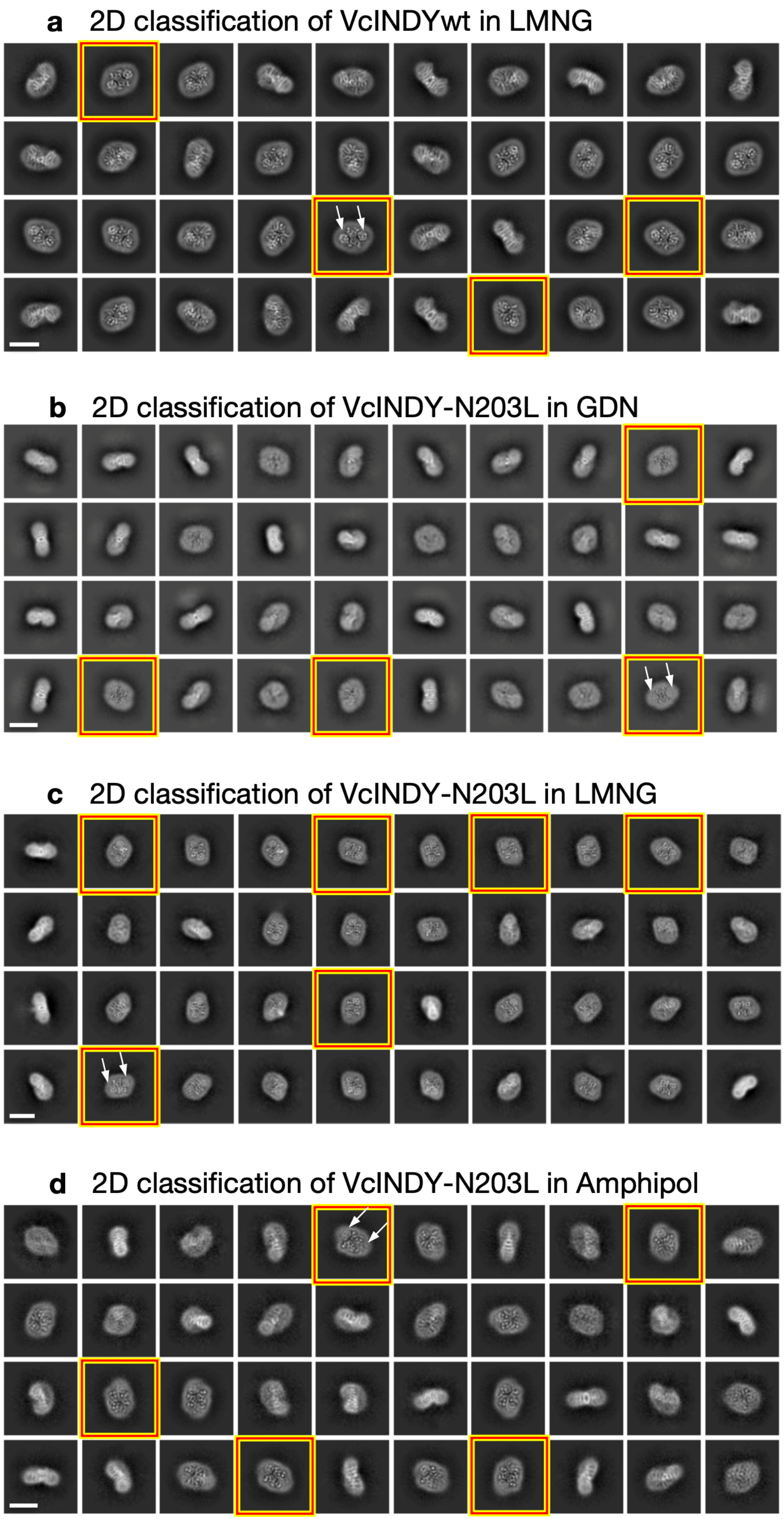

Supplementary Fig. 10. 2D classification of VcINDY in various detergents. a, VcINDYwt in LMNG. In top views (perpendicular to the membrane, as indicated by red/yellow squares), both the scaffold domains at the dimer interface and the transport domains in each protomer are clearly

resolved. Individual white densities represent the projections of helices perpendicular to the membrane. The helices in each transport domain form a circular shape. **b**, VcINDY-N203L in LMNG. **c**, VcINDY-N203L in GDN. **d**, VcINDY-N203L in Amphipol. In top views of the VcINDY-N203L mutant in **b-d**, only the scaffold domains are resolved, whereas the transport domains are largely blurred. The scale bars represent 100 Å. Representative top views are indicated in red/yellow boxes, and the two white arrows point to the transport domains in the dimer.

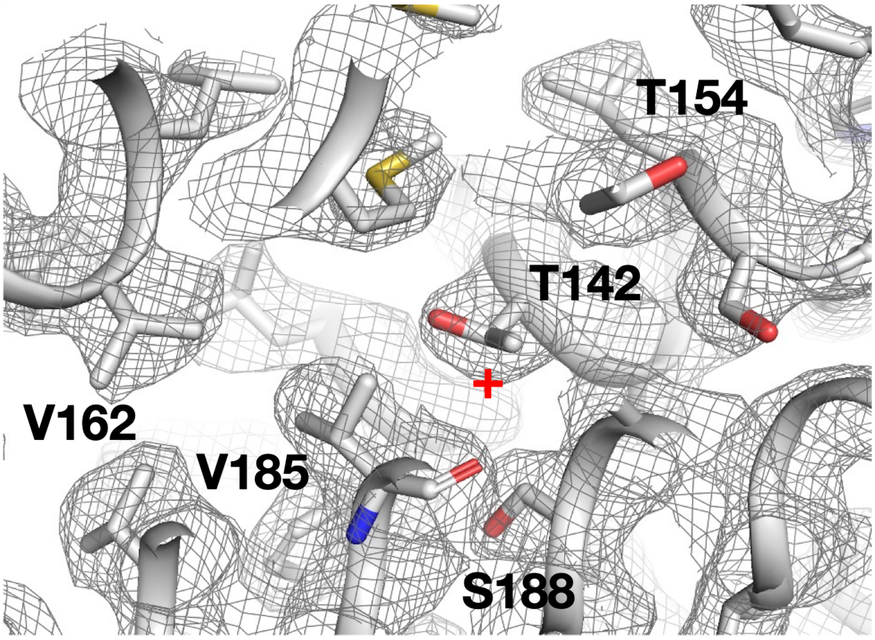

Supplementary Fig. 11. Local cryo-EM map of wildtype VcINDY. Our cryo-EM map for VcINDYwt is shown for the position of the new metal (M) site identified in the SiaQM protein from *Haemophilus influenzae*[7]. The location of the M site is indicated with a red "+" sign. No density is visible at the location**.** The map is contoured at 5.5 σ.

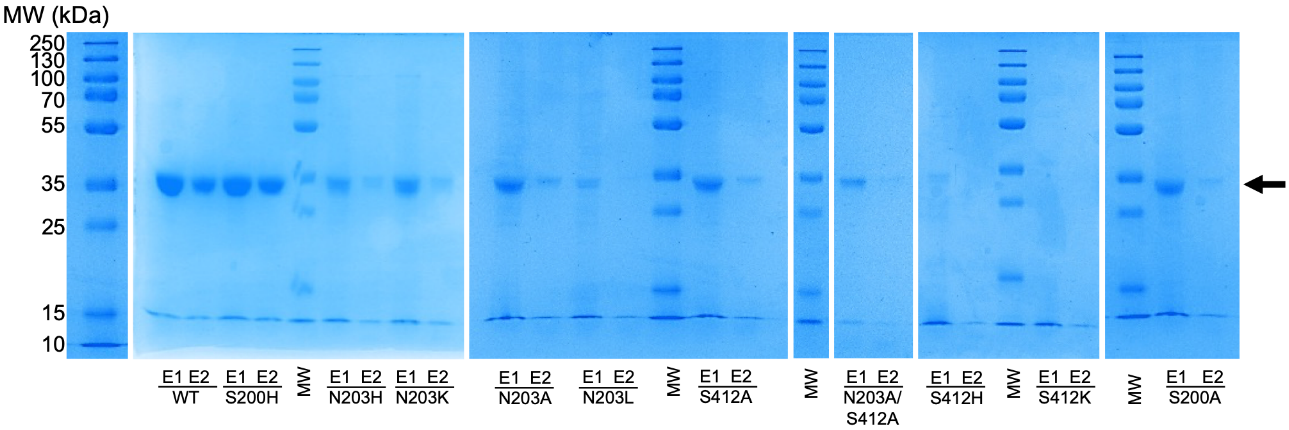

Supplementary Fig. 12. SDS-PAGE analysis of IMAC-purified VcINDY variants used in this study. The gel depicts the 2 IMAC elution fractions for VcINDY wildtype (WT) and each of the variants used during this study; S200H, N203H, N203K, N203A, N203L S412A, N203L:S412A double mutant, S412H, S412K and S200A. The black arrow indicates the protein band corresponding to VcINDY. Each construct was purified on more than one occasion with similar results, except S412H and S412K, which were only tested a single time. MW is the molecular weight ladder used; the molecular weight of each band is indicated on the left-hand ladder.

Supplementary References

1. Mancusso, R., Gregorio, G. G., Liu, Q. & Wang, D. N. Structure and mechanism of a bacterial sodium-dependent dicarboxylate transporter. *Nature* **491**, 622–626 (2012).
2. Nie, R., Stark, S., Symersky, J., Kaplan, R. S. & Lu, M. Structure and function of the divalent anion/Na+ symporter from Vibrio cholerae and a humanized variant. *Nature Communications* **8**, 15009 (2017).
3. Kinz-Thompson, C. D. *et al.* Elevator mechanism dynamics in a sodium-coupled dicarboxylate transporter. *Proceedings of the National Academy of Sciences* **123**, e2500723123 (2026).
4. Sauer, D. B. *et al.* Structural basis for the reaction cycle of DASS dicarboxylate transporters. *eLife* **9**, 213 (2020).
5. Sauer, D. B. *et al.* Structural basis of ion - substrate coupling in the Na+-dependent dicarboxylate transporter VcINDY. *Nat Communications* **13**, 2644 (2022).
6. Madeira, F. *et al.* The EMBL-EBI Job Dispatcher sequence analysis tools framework in 2024. *Nucleic Acids Res* **52**, W521–W525 (2024).
7. Goyal, P. *et al.* Molecular determinants of Neu5Ac binding to a tripartite ATP independent periplasmic (TRAP) transporter. *eLife* **13**, RP98158 (2024).
